# Rapid Photogelation of Low‐Concentration Polymer Solutions via Photodimerization of *o*‐Ethylnitrobenzene

**DOI:** 10.1002/advs.77119

**Published:** 2026-08-31

**Authors:** Masaaki Okihara, Ayaka Tomoda, Kana Morishita, Toshiyuki Takagi, Kimio Sumaru

**Affiliations:** ^1^ Cellular and Molecular Biotechnology Research Institute National Institute of Advanced Industrial Science and Technology Tsukuba Ibaraki Japan

**Keywords:** in‐gel cell culture, photocrosslinking, photodimerization, sol–gel transition

## Abstract

Polymers that exhibit significant property changes upon light stimulation are promising for biomaterials due to their minimally invasive nature and spatiotemporal controllability. In this study, *o*‐ethylnitrobenzene (ENB) was identified as an effective photocrosslinking motif and incorporated into poly(vinyl alcohol) (PVA), yielding a new class of photocrosslinkable, water‐soluble polymers. These polymers undergo rapid gelation (∼0.5 s) from dilute aqueous solutions (∼0.5 wt.%) upon UV irradiation to form flexible and elastic hydrogels. The resulting hydrogels exhibit high compressive resilience; for example, a cylindrical hydrogel prepared from a 1.5 wt.% solution withstands a compressive stress exceeding 10 MPa without fracture. Hydrogels formed from 2 wt.% solutions swell isotropically up to 200 fold in water while retaining sufficient mechanical strength for manual handling, even at a water content of 99.99 wt.%. Furthermore, the addition of the polymer to cell‐containing media, followed by photoinduced gelation in the presence of cells, preserves cell viability. Cells are instantaneously immobilized in 3D space within the hydrogel, forming a highly permeable culture system that enables long‐term culture through medium exchange and promotes the formation of large cellular spheroids. These results highlight the potential of ENB‐based hydrogels for spatiotemporally controlled 3D cell culture applications.

## Graphical abstract

Upon UV irradiation, flexible and tough hydrogels are formed rapidly (>0.5 s) from dilute (>0.5 wt%) solutions of *o‐*ethylnitrobenzene–modified PVA. At ∼1.5 wt%, the hydrogels withstand up to 10 MPa compression, remain handleable even after 200‐fold swelling. 3D cell culture maintaining cell viability stably in hydrogel is instantly implemented just by irradiating UV to the polymer‐containing cell suspensions.

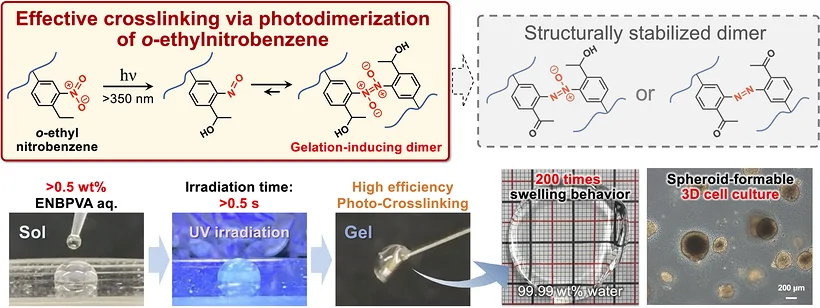

## 1 Introduction

The sol–gel transition is a phase transition observed in many synthetic and natural polymers, in which a liquid solution (sol) transforms into a gel with a 3D network structure. Various gelation mechanisms for polymer solutions have been developed, including photo‐crosslinking, click chemistry, condensation reactions, and enzymatic crosslinking. Because these systems are based on fundamentally different crosslinking principles, they exhibit distinct characteristics in terms of gelation trigger, gelation kinetics, spatiotemporal controllability, and bioorthogonality (Table S1). Therefore, they should be discussed and compared according to their respective characteristics rather than solely on the basis of reaction efficiency or mechanical properties.

Among these gelation mechanisms, click chemistry‐based gelation has attracted considerable interest owing to its high reaction rates and excellent selectivity. Representative examples include azide–alkyne cycloaddition, thiol–ene reactions, and Diels–Alder cycloadditions [1, 2]. However, such systems generally require the mixing of multiple components bearing reactive functional groups, which makes precise spatial and temporal control over reaction initiation inherently difficult.

Stimuli‐responsive polymers, including photo‐crosslinkable polymers, have attracted considerable attention because their sol‐to‐gel transitions can be precisely regulated by external stimuli such as temperature, pH, chemical species, and light [3, 4, 5, 6, 7, 8, 9, 10, 11]. Among them, light‐triggered sol–gel transitions are particularly appealing because irradiation can induce the response at precisely defined positions and times, thereby enabling exceptional spatiotemporal control. In particular, photo‐crosslinkable polymers that do not require additional reagents have been highly attractive due to their minimally invasive nature and permit precise material fabrication as well as manipulation in cellular environments. Accordingly, they have emerged as important materials for research in the fields of biomaterials and photoengineering.

**FIGURE 1:**
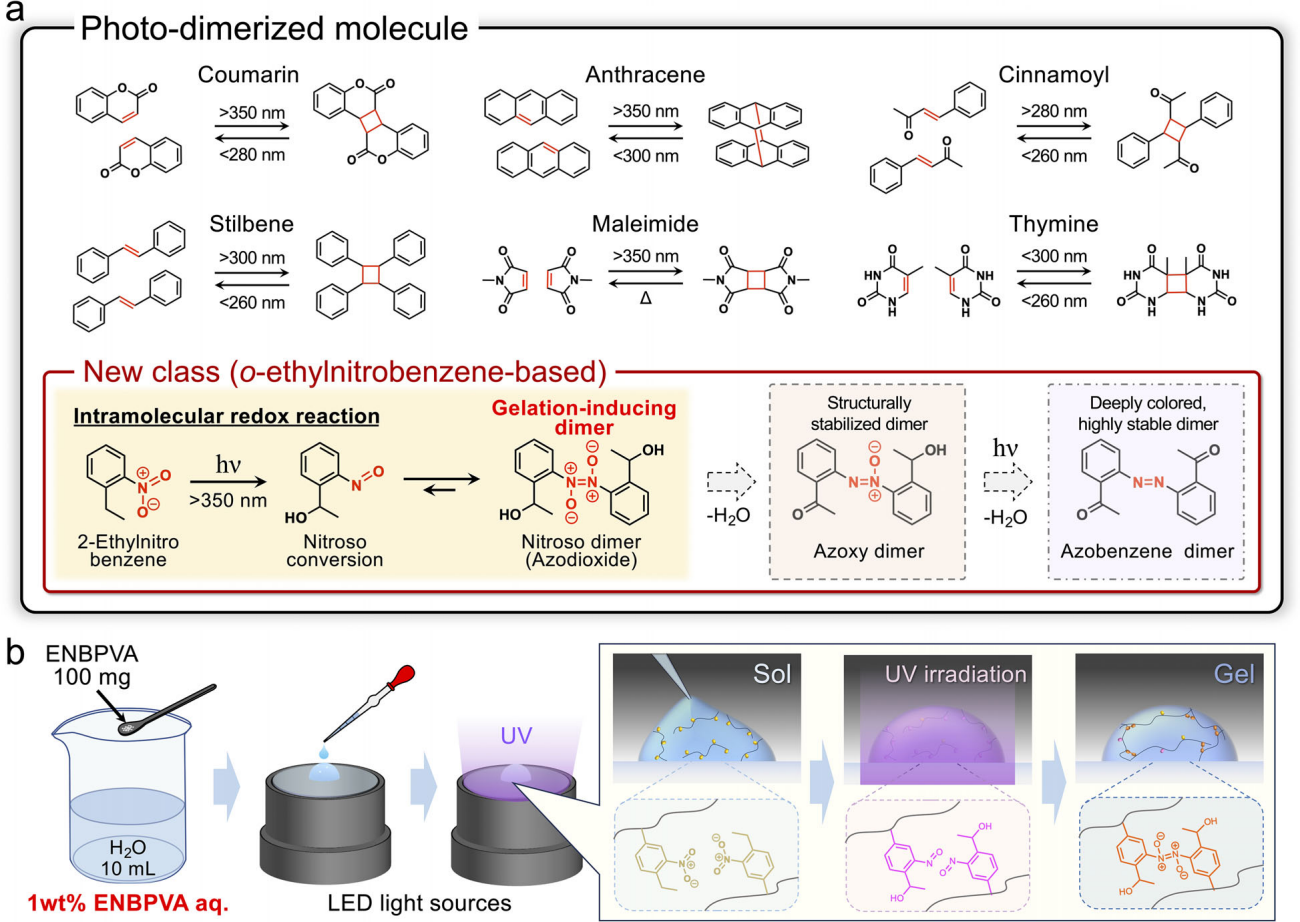
(a) A list of previously reported photodimerizable molecules, including *o*‐ethylnitrobenzene, which is proposed as a new class of photodimerizable compound. (b) Schematic illustration of the photogelation process of ENBPVA.

Representative photodimerization motifs that have been introduced into polymer side chains include cinnamoyl [12, 13], anthracene [14, 15], coumarin [16, 17], maleimide [13, 18], stilbene [19], and thymine [20] (Figure 1a), and some of them have already been commercialized. However, most photocrosslinkable polymers incorporating these photoresponsive groups require high polymer concentrations (>10 wt.%) to form hydrogels with sufficient mechanical strength. In addition, because concentrated polymer solutions generally exhibit reduced light transmittance, gelation often becomes insufficient at depths greater than ∼1 mm from the irradiated surface. By contrast, hydrogel formation from dilute polymer solutions has been reported for various chemical and physical gelation systems. Nevertheless, achieving rapid gelation solely upon light irradiation while maintaining high mechanical robustness under dilute aqueous conditions remains challenging.

Like other photo‐controllable gelation systems, photopolymerization based on macromonomers has been extensively studied and widely used to construct cell culture systems [21, 22]. Gelatin methacryloyl (GelMA) and polyethylene glycol diacrylate (PEGDA) have especially been used as radical‐polymerizable macromonomers in combination with photo‐initiators such as lithium phenyl(2,4,6‑trimethylbenzoyl)phosphinate (LAP), which generate radical species upon light irradiation.

Under these circumstances, we observed a phenomenon that motivated this study in our previous work on a polymer bearing *o*‐nitrobenzaldehyde (NBA) side chains, which exhibited pronounced photoinduced dissolution from a water‐insoluble state [23]. In a synthetic intermediate where the aldehyde group of NBA was protected by acetalization, we obtained some observations suggestive of photocrosslinking. We hypothesized that this phenomenon arose from the dimerization of nitroso groups generated upon photoirradiation, a reaction mechanism that has long been recognized [24, 25, 26, 27, 28]. On the basis of this insight, we examined various aromatic nitro groups and found that *o*‐ethylnitrobenzene (ENB), whose photoresponsive behavior has not previously been investigated, exhibited excellent photocrosslinking properties.

In this study, we introduced ENB into the side chains of polyvinyl alcohol (PVA) via acetalization to afford a water‐soluble polymer, ENBPVA, and examined its photocrosslinking behavior in aqueous solution, with particular emphasis on its photosensitivities, light transmittance in solution and gel states, and gelation ability at low concentrations (Figure 1b). We further evaluated the properties of ENBPVA hydrogels formed by photocrosslinking, including resistance to compressive failure and mechanical flexibility, to assess their potential as high‐performance soft materials. We also investigated its potential as a photocontrollable biotool for 2D/3D in vitro cell culture applications.

## 2 Results and Discussion

### 2.1 Synthesis and Photocrosslinking Behavior of ENBPVA

**FIGURE 2:**
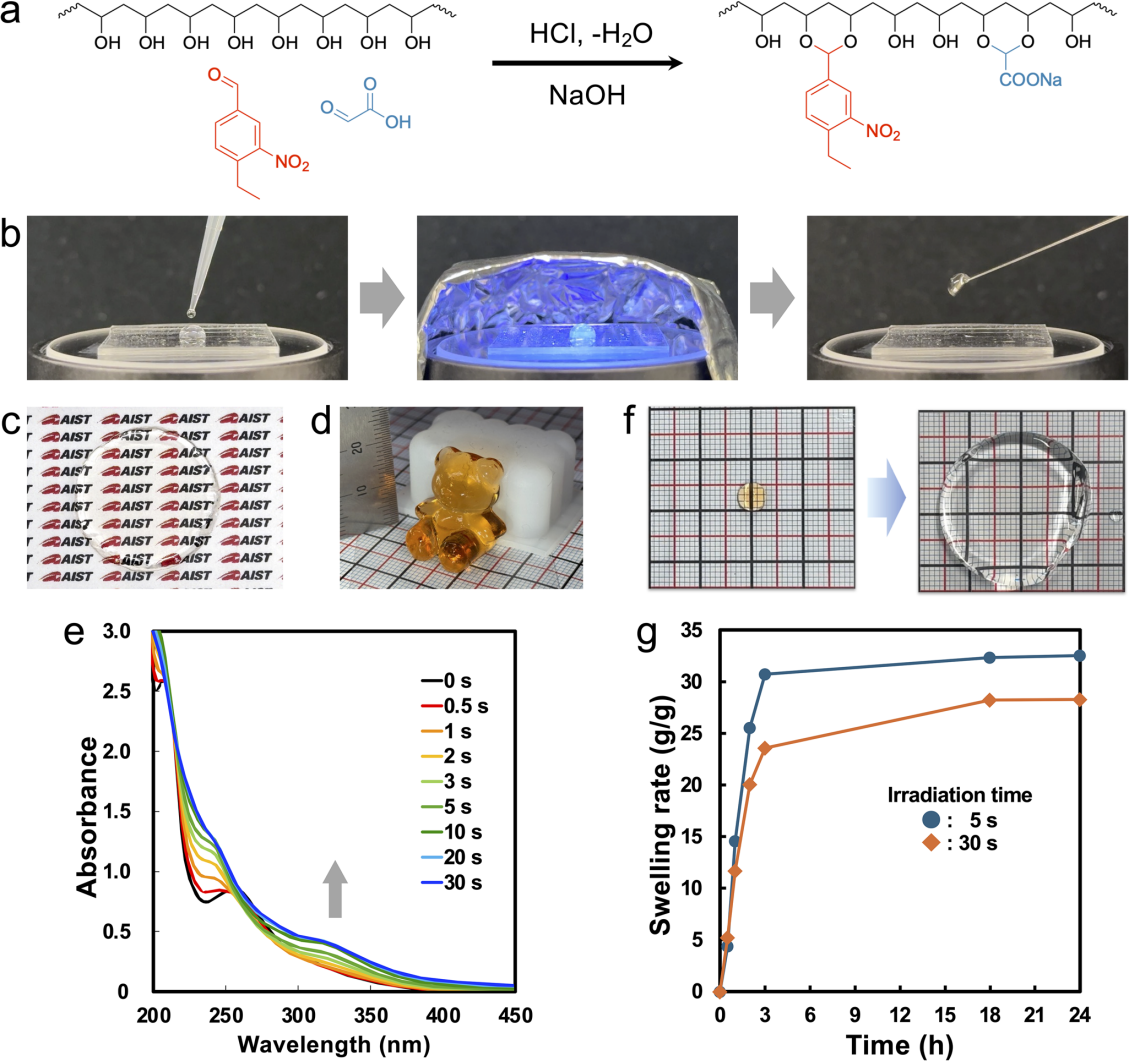
(a) Synthetic scheme of ENBPVA. (b) Sequential photographs showing the gelation behavior of a 2 wt.% ENBPVA solution upon irradiation with 365 nm UV light (240 mW cm^−^^2^) for 1 s. (c) Photograph demonstrating the high transparency of the hydrogel (10 mm diameter, 1 mm height) formed by irradiating a 2 wt.% ENBPVA solution with 365 nm UV light for 5 s. (d) Photograph of a bear‐shaped hydrogel prepared by pouring a 1.5 wt.% ENBPVA solution into a bear‐shaped silicone mold and irradiating it with 365 nm UV light. (e) UV–vis spectra of ENBPVA in water after UV irradiation (365 nm, 240 mW cm^−^^2^) for various periods. The concentration of ENBPVA in aqueous solution was 0.1 wt.%. (f) Photographs of a hydrogel before and after swelling in ultrapure water for 1 day. The hydrogel was prepared by irradiating a 2 wt.% ENBPVA solution with UV light (365 nm, 240 mW cm^−^^2^) for 5 s. (g) Swelling ratio of hydrogels prepared by UV irradiation (365 nm, 240 mW cm^−^^2^) for 5 or 30 s.

The synthetic scheme and molecular structure of ENBPVA are shown in Figure 2a. NMR analysis confirmed that the main chain of high‐molecular‐weight PVA was functionalized with 0.59 mol% ENBA and 2.01 mol% carboxyl groups, relative to the PVA repeating units, via acetal linkages to hydroxyl groups (Figure S1). The introduction of carboxyl groups into ENBPVA serves to improve the water solubility, which is reduced by the incorporation of hydrophobic ENB groups, and to promote intermolecular crosslinking by stretching the polymer chains through electrostatic repulsion. Hydrogel formation upon light irradiation of a droplet of ENBPVA aqueous solution is shown in Figure 2b and Movie S1. When 20 µL of 2 wt.% ENBPVA solution was irradiated with UV light at 365 nm (240 mW cm^−^^2^) for 5 s, it was rapidly converted into a highly elastic hydrogel. The resulting ENBPVA hydrogel exhibited excellent transparency and high visible light transmittance (Figure 2c and Figure S2). Owing to the high transmittance of ENBPVA solutions, hydrogels with arbitrary shapes on the centimeter scale can be fabricated by a single light exposure. Figure 2d shows a hydrogel object formed by photocrosslinking 2.5 mL of 1.5 wt.% ENBPVA solution in a silicone mold with a depth of up to 13 mm. This object, composed of 98.5 wt.% water, maintained the mold shape without significant deformation under its own weight (∼2.5 g), indicating sufficient light penetration and crosslinking even at such depth. After prolonged light irradiation, the ENBPVA hydrogel turned a light orange color, suggesting the generation of an azobenzene‐like structure, which will be discussed in detail later. However, as shown in Figure 2b,c, short irradiation times yield a colorless hydrogel, indicating that the nitroso dimer itself is responsible for crosslink formation essential for gelation. Figure 2e shows the change in UV–vis spectra of 0.1 wt.% ENBPVA solution (path length: 10 mm) upon irradiation with the same UV light. These results suggested that most ENB moieties underwent a photoreaction during the first 5 s of irradiation, indicating the system's high sensitivity to light. The absorbance remained within the 0.1–0.2 range at 365 nm (irradiation wavelength), indicating that light of sufficient intensity penetrates deeply (centimeter‐scale) even at concentrations of 1–2 wt.%. This supports the feasibility of fabricating large hydrogel structures, such as those shown in Figure 2d, via single‐step photoirradiation. Furthermore, most ENB groups were photochemically converted within 10 s of irradiation, confirming their high sensitivity to light at this wavelength.

The ENBPVA hydrogel also swelled isotropically to more than 10 000%, maintaining its original shape at equilibrium without breakage or dissolution (Figure 2f and Movie S2). The swelling behavior of the hydrogel prepared from 2 wt.% ENBPVA is shown in Figure 2f. Even after swelling up to 200 times its original volume and reaching 99.99 wt.% water content, the hydrogel retained sufficient mechanical strength to be handled by hand (Movie S3). These results indicate that the crosslinks formed upon irradiation of the ENB motif and the structure of the polymer network in ENBPVA hydrogel were highly stable. Figure 2g shows the time‐dependent swelling behavior of photo‐crosslinked ENBPVA hydrogels in pure water. A hemispherical hydrogel (diameter: 5 mm), prepared from 2 wt.% ENBPVA solution, reached swelling equilibrium in ∼3 h. The degree of swelling decreased with increasing UV irradiation time, likely due to higher crosslink density from more extensive photocrosslinking. Based on the results described above, this approach can provide an effective method for preparing low‐concentration gels exhibiting excellent mechanical performance comparable to so‐called Aquamaterials [29] and polyisocyanopeptide (PIC) gels [30] reported by Aida et al. and by Rowan et al., respectively.

**FIGURE 3:**
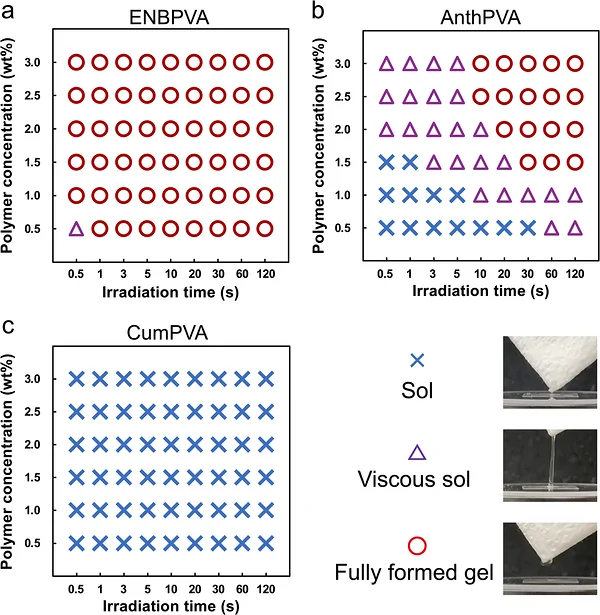
Phase diagrams showing the dependence of photogelation behavior on polymer concentration and irradiation time for aqueous solutions of (a) ENBPVA, (b) AnthPVA, and (c) CumPVA under 365 nm irradiation. The states of sol (×), viscous sol (△), and fully formed gel (○) were determined based on the behavior upon contact with a wipe, as shown in the corresponding photographs.

Photocrosslinkable PVAs incorporating anthracene (Anth) and coumarin (Cum), which are established photodimerization motifs responsive to 365 nm irradiation, were synthesized (AnthPVA and CumPVA) and used as comparators for ENBPVA. ^1^H NMR analysis confirmed that AnthPVA contained 0.66 mol% Anth and 3.5 mol% carboxyl groups relative to vinyl alcohol units (Figure S3). Similarly, CumPVA was found to contain 0.64 mol% Cum and 2.5 mol% carboxyl groups (Figure S4). AnthPVA synthesized with a composition comparable to that of ENBPVA exhibited poor water solubility, with most of the material precipitating, making it difficult to prepare a homogeneous solution. This behavior is attributed to the significantly higher hydrophobicity and aggregation tendency of the Anth moiety compared with ENB, leading to strong hydrophobic interactions in aqueous media and consequent aggregation of Anth moieties. To improve the water solubility of AnthPVA, a 1.5 fold increase in carboxyl group content was introduced during synthesis. Figure 3 presents a phase diagram illustrating the influence of both polymer concentration and irradiation time on the photogelation of the aqueous solutions of (a) ENBPVA, (b) AnthPVA, and (c) CumPVA, respectively. The states of sol (×), viscous sol (△), and fully formed gel (○) were determined by visually observing the behavior upon contact with a wipe (Figure S5). For AnthPVA, hydrogels were fully formed at polymer concentrations greater than 1.5 wt.% by irradiation for 30 s or longer. In contrast, for CumPVA, only a viscosity increase was observed within the polymer concentration range up to 3 wt.%, even upon prolonged irradiation, and no distinct hydrogel formation occurred. Gelation of CumPVA required 5 wt.% polymer concentration and 300 s of irradiation. By comparison, ENBPVA formed a complete hydrogel at a polymer concentration of 1 wt.% with only 0.5 s of irradiation. Furthermore, gelation was also observed at 0.5 wt.% with 1 s of irradiation. These results indicated that the photocrosslinking by the ENB moiety is much more efficient than that of conventional photodimerization motifs such as Anth or Cum.

### 2.2 Mechanical Property Evaluation of ENBPVA Hydrogels

**FIGURE 4:**
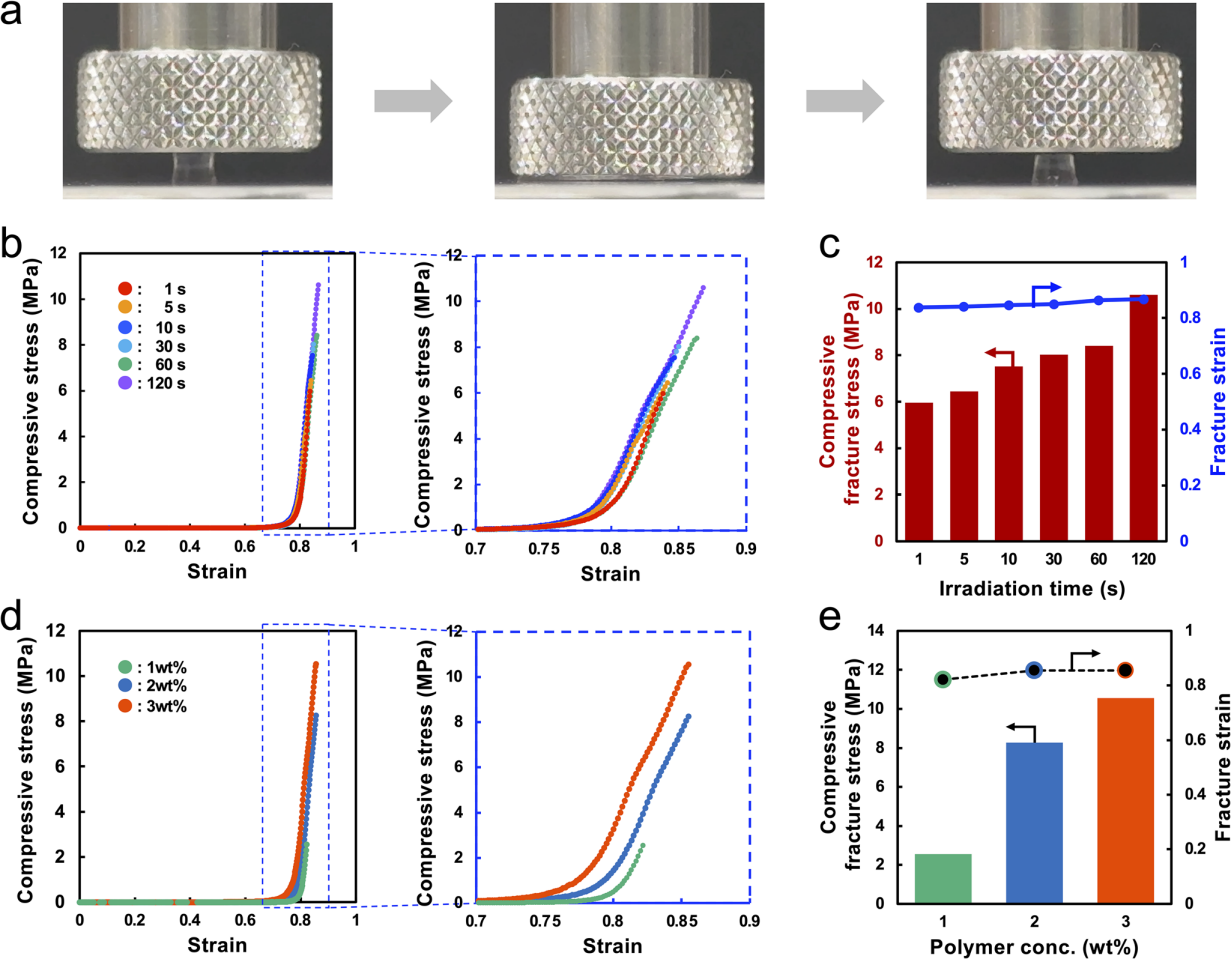
(a) Compression test of hydrogels prepared by pouring a 1.5 wt.% ENBPVA solution into a cylindrical silicone mold (3 mm diameter, 2 mm height) and irradiating with 365 nm UV light (240 mW cm^−^^2^) for 1 s; sequential photographs during compression. (b) Stress–strain curves (left) of 1.5 wt.% ENBPVA hydrogels prepared with various UV irradiation times (365 nm, 240 mW cm^−^^2^). The corresponding curves (right) are enlarged in the strain range of 0.7–0.9. (c) Fracture compressive stress and strain of ENBPVA hydrogels as a function of UV irradiation time, extracted from the stress–strain curves in Figure 4b. (d) Stress–strain curves (left) of ENBPVA hydrogels prepared at different ENBPVA concentrations with a fixed UV irradiation condition (365 nm, 240 mW cm^−^^2^, 10 s). The corresponding curves (right) are enlarged in the strain range of 0.7–0.9. (e) Fracture compressive stress and strain of ENBPVA hydrogels as a function of ENBPVA concentration, extracted from the stress–strain curves in Figure 4d.

The mechanical properties of the photo‐crosslinked ENBPVA hydrogels were evaluated by compression testing (Figure 4a) using cylindrical specimens (3 mm diameter, 2 mm height). Compressive stress was calculated using the initial cross‐sectional area (as discussed later). Stress–strain curves of hydrogels prepared from 1.5 wt.% ENBPVA solutions under varying irradiation doses are shown in Figure 4b. In all cases, the hydrogels exhibited minimal stress response until compressed to 75% strain, indicating highly compliant mechanical behavior. Beyond this strain, stress increased sharply, reaching a maximum fracture stress of 10.4 MPa at the longest irradiation time (Figure 4c). The compressive fracture stress increased with irradiation time, suggesting an increase in crosslinking density reflecting enhanced load‐bearing capacity of the network.

In general, the crosslink density of a hydrogel can be estimated from its elastic modulus in the low‐strain region using Equation (3) shown below in the Experimental Section [31, 32]. For the 1.5 wt.% ENBPVA hydrogel, the effective crosslink density ν*_eff_* was thus calculated from the mechanical properties of the hydrogel prepared under varying light exposure times (Figure S6). On the other hand, the maximum chemical crosslink density ν*_ch, max_* can also be calculated from the ENB incorporation rate. In the case where all the ENB groups incorporated in ENBPVA at rate of 0.59 mol% are fully dimerized, ν*_ch, max_* is estimated to be 1.0 mol/m^3^. The ν*_eff_* value rapidly increased in the first second of light irradiation, while a gradual increase was observed during the subsequent 60 s of irradiation. This suggests that the crosslinking process is highly light‐sensitive but becomes increasingly hindered as it progresses, due to the difficulty for ENB groups to find nearby counterparts for crosslinking. Such kinetic processes are also supported by data showing the influence of irradiation time on fracture compressive stress (Figure 4c). Remarkably, even a hydrogel formed by only 1 s of UV exposure showed a fracture stress of 5.9 MPa. A hemispherical specimen (0.05 g), prepared by 0.5 s irradiation of 3 wt.% ENBPVA solution, withstood 30 N (3 kgf) compressive force without breaking (Movie S4).

**FIGURE 5:**
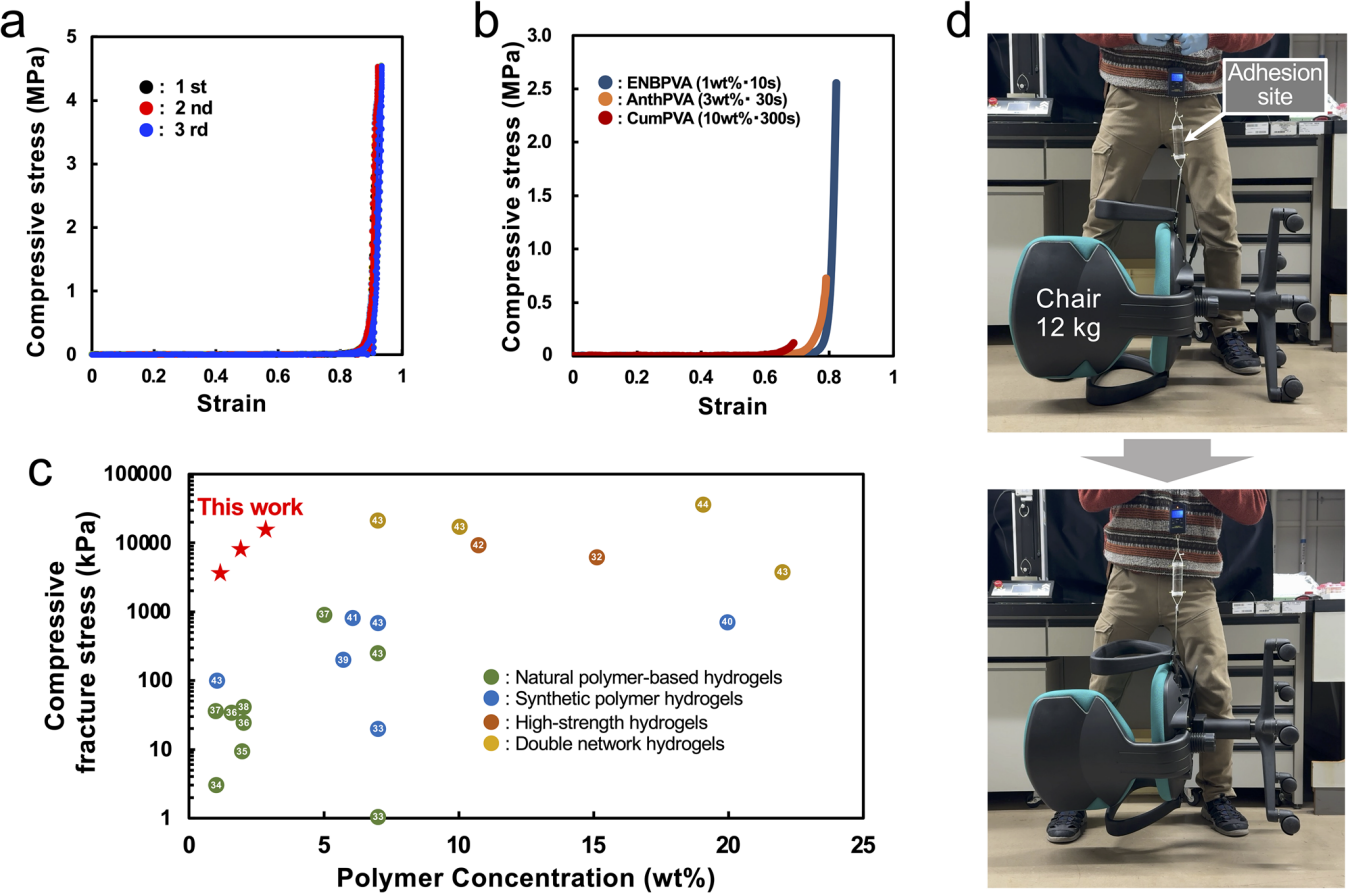
(a) Stress–strain curves of 1.5 wt.% ENBPVA hydrogels under repeated compression. The hydrogel was prepared by UV irradiation (365 nm, 240 mW cm^−^^2^) for 1s. (b) Compressive stress–strain curves of hydrogels prepared from AnthPVA, CumPVA, and ENBPVA under their respective minimum gelation conditions required to form cylindrical specimens. The polymer concentrations and irradiation times were 1 wt.% and 10 s for ENBPVA, 3 wt.% and 30 s for AnthPVA, and 10 wt.% and 300 s for CumPVA. (c) Relationship between polymer concentration and fracture compressive stress for previously reported hydrogels and ENBPVA hydrogels. A list of the hydrogels used for comparison is provided in Table S2 [32, 33, 34, 35, 36, 37, 38, 39, 40, 41, 42, 43, 44]. Red stars represent ENBPVA hydrogels developed in this study. Green circles represent Natural polymer‐based hydrogels, blue circles represent synthetic polymer hydrogels, orange circles represent “high‐strength” hydrogels, and yellow circles represent double network hydrogels. The numbers in the graphs correspond to the reference numbers cited. (d) Photograph of a 12 kg chair being lifted by hand using two cylindrical acrylic rods bonded with ENBPVA hydrogel as connectors. The rods were bonded by sandwiching a 3 wt.% ENBPVA solution between them, followed by UV irradiation (365 nm, 240 mW cm^−^^2^) for 40 s.

Figure 4d shows the dependence of mechanical properties on polymer concentration. Compressive fracture stress increased with increasing ENBPVA concentration. Even at 1 wt.%, the hydrogel achieved a compressive fracture stress exceeding 2 MPa (Figure 4e). When 1.5 wt.% ENBPVA hydrogels prepared with 1 s irradiation were subjected to repeated compressive loading at 4.5 MPa for three cycles, the stress–strain curves remained nearly identical (Figure 5a), indicating structural stability under repeated stress. Figure 5b presents a comparative analysis of the compressive responses of hydrogels formed from AnthPVA, CumPVA, and ENBPVA. The gelation conditions for AnthPVA and CumPVA were selected as the minimum polymer concentration and shortest irradiation time at which cylindrical specimens (3 mm in diameter and 2 mm in height) could be reproducibly formed for compression testing. Under these conditions, the compressive fracture stress of the AnthPVA hydrogel formed at a polymer concentration of 3 wt.% with 30 s of irradiation was 0.72 MPa. In contrast, the CumPVA hydrogel formed at a polymer concentration of 10 wt.% with 300 s of irradiation exhibited a compressive fracture stress of 0.11 MPa. By comparison, the ENBPVA hydrogel formed at a polymer concentration of 1 wt.% with only 10 s of irradiation exhibited a higher compressive fracture stress of 2.55 MPa. These results indicate that ENBPVA hydrogels exhibit superior resistance to compression under large deformation conditions compared to hydrogels formed via conventional photodimerization motifs, suggesting enhanced structural integrity of the network.

Swollen ENBPVA hydrogels were prepared by irradiating a 2 wt.% ENBPVA solution with light for 10 s and subsequently immersing the hydrogels in ultrapure water for varying periods. The mechanical properties of these swollen hydrogels were evaluated via compression testing. The compressive fracture stress at different degrees of swelling is compared in Figure S4. It was found that swelling substantially reduces the compressive fracture stress of ENBPVA hydrogels. This reduction is attributed to the low polymer concentrations under highly hydrated conditions (0.5 wt.% at fourfold swelling and 0.057 wt.% at 35 fold swelling), which decreases the stability of the network structure and diminishes energy dissipation arising from noncovalent interactions such as chain entanglements. However, it should be noted that the compressive fracture stress was still measurable under such low polymer content conditions and that the swollen hydrogel with 99.99 wt.% water content retained sufficient mechanical strength to be handled manually (Figure 2f and Movie S3).

A comparative analysis of the relationship between polymer concentration and compressive fracture stress for various previously reported hydrogels [32, 33, 34, 35, 36, 37, 38, 39, 40, 41, 42, 43, 44] revealed that ENBPVA hydrogels exhibit the highest compressive fracture stress in the low‐concentration regime (Figure 5c and Table S2). Notably, ENBPVA exhibits an atypical compressive response in that, despite its extremely high water content of up to 99 wt.%, it shows a low initial elastic modulus while maintaining structural integrity without collapse even in the large compressive deformation regime. It should be noted that the compressive fracture stress discussed in this study, including the previously reported results referred to above, is based on “engineering stress” calculated by using the initial cross‐sectional area as described above. In such highly flexible systems, the hydrogel undergoes substantial deformation, leading to expansion of the cross‐sectional area prior to fracture, and we should be careful not to overestimate the mechanical strength of the hydrogel. This situation is rather general due to the difficulty of monitoring the cross‐sectional area of gels during compression testing. Nevertheless, the cross‐sectional area immediately prior to fracture was estimated to be 177 mm^2^ from the image analysis of the movie data recorded for the hydrogel formed from a 3 wt.% polymer solution by 10 s irradiation. Considering the initial area had been 7.1 mm^2^, the practical compressive fracture stress is calculated to be ∼0.4 MPa.

Although tensile characterization is important for evaluating the mechanical properties of hydrogels, accurate measurements are often challenging for highly hydrated and soft hydrogels because stable fixation of the specimens is difficult and fracture frequently occurs at the grips rather than within the bulk gel [45, 46, 47]. ENBPVA hydrogels exhibited similarly high flexibility and deformability, making it difficult to accurately determine their intrinsic tensile strength using conventional tensile testing methods. Therefore, as a demonstration of the tensile properties of the hydrogel under conditions where no significant deformation occurs, adhesion arising from the photoresponsive sol–gel transition was also evaluated. A 3 wt.% ENBPVA solution was placed between the end faces of two PMMA resin cylinders (φ30 mm) and irradiated with UV light to induce gelation. The resulting hydrogel enabled adhesion sufficient to suspend a 12 kg load (Figure 5d and Movie S5). Tensile testing confirmed an adhesive strength of 140 N (Figure S8 and Movie S6), and the load‐bearing capacity was estimated to be 0.2 MPa based on the cross‐sectional area of the PMMA cylinders. Among several plastics, PMMA was selected because it exhibits strong interfacial interactions with the ENBPVA hydrogel, making it suitable for evaluating the fracture resistance of the hydrogel. Under vertical tensile deformation, fracture occurred predominantly within the hydrogel bulk rather than at the PMMA–hydrogel interface, as hydrogel residues remained on both PMMA surfaces after fracture. Furthermore, because the area attached to the PMMA cylinders was much larger than the thickness of the hydrogel layer, bulk failure occurred without significant deformation of the hydrogel. In this configuration, however, it is necessary to consider the suction effect arising from atmospheric pressure (∼0.1 MPa). After deducting this contribution, the tensile strength was estimated to be ∼0.1 MPa. Although this value should not be directly compared with those obtained by conventional tensile testing using standardized specimens, it demonstrates that the highly hydrated ENBPVA hydrogel (97 wt.% water) can withstand substantial tensile stress under adhesive loading conditions.

To investigate the mechanism underlying this unique mechanical property, we examined the effect of UV intensity on photocrosslinking. Under dilute conditions that do not result in macroscopic gelation, the hydrodynamic diameter of light‐induced polymer clusters was measured by dynamic light scattering (DLS). Results showed that lower photon flux led to the formation of larger ENBPVA clusters (Figure S9a), suggesting a mechanism in which ENB units are sequentially photoconverted to an active and relatively stable dimerizable form (ENB*). At low photon flux, the generation rate of ENB* is significantly suppressed, so there tends to be only one ENB* per polymer chain. Such a situation is considered to reduce the likelihood of intramolecular dimerization, favoring intermolecular crosslinking with ENB* on other chains. The preferential formation of inter‐chain crosslinks results in a more effective network structure and enhanced gelation efficiency. (Figure S9b).

### 2.3 Photocrosslinking Mechanism of ENB Groups

To date, no reports have described the photochemistry of the ENB structure, which we have identified in this study as a novel photocrosslinking motif. As mentioned above, photocrosslinking was first observed in a polymer functionalized with an acetal‐protected derivative of *o*‐nitrobenzaldehyde [23], a compound known to undergo photoconversion to dimerizable nitroso derivatives. This suggested that a similar photochemical pathway may underlie the photocrosslinking behavior of ENB [24, 25, 26, 27, 28]. To test this hypothesis, we first examined a mixed aqueous solution of PVA and 4‐ethyl‐3‐nitrobenzoic acid (ENBAc), a water‐soluble small molecule bearing the ENB group (Figure S10). No change in physical properties was observed upon photoirradiation, indicating that the photocrosslinking reaction requires covalent attachment of ENB to the polymer backbone and is not due to a remote effect such as photosensitization of PVA by irradiated ENB groups. Next, to gain insight into the role of the ENB group in the photocrosslinking of ENBPVA, we investigated the effect of the coexistence of ENBAc as a monomeric ENB species. As a result, the photo‐induced gelation of ENBPVA aqueous solution was effectively prevented by the addition of ENBAc (Movie S7). The results suggested that the ENB moieties on the side chain of ENBPVA preferentially underwent photodimerization with monomeric ENBAc capable of moving freely, thereby effectively inhibiting intermolecular crosslinking between polymer chains. In contrast, benzoic acid—a compound with a similar structure—had no influence on the photo‐induced gelation of ENBPVA aqueous solution. To investigate whether radical species are involved in the reaction, experiments were conducted using the well‐known radical scavenger 2,2,6,6‑tetramethylpiperidin‑1‑oxyl (TEMPO). However, the addition of TEMPO had no influence on the gelation (Figure S11). All these findings strongly support the conclusion that photocrosslinking in ENBPVA proceeds via photodimerization between ENB side chains.

**FIGURE 6:**
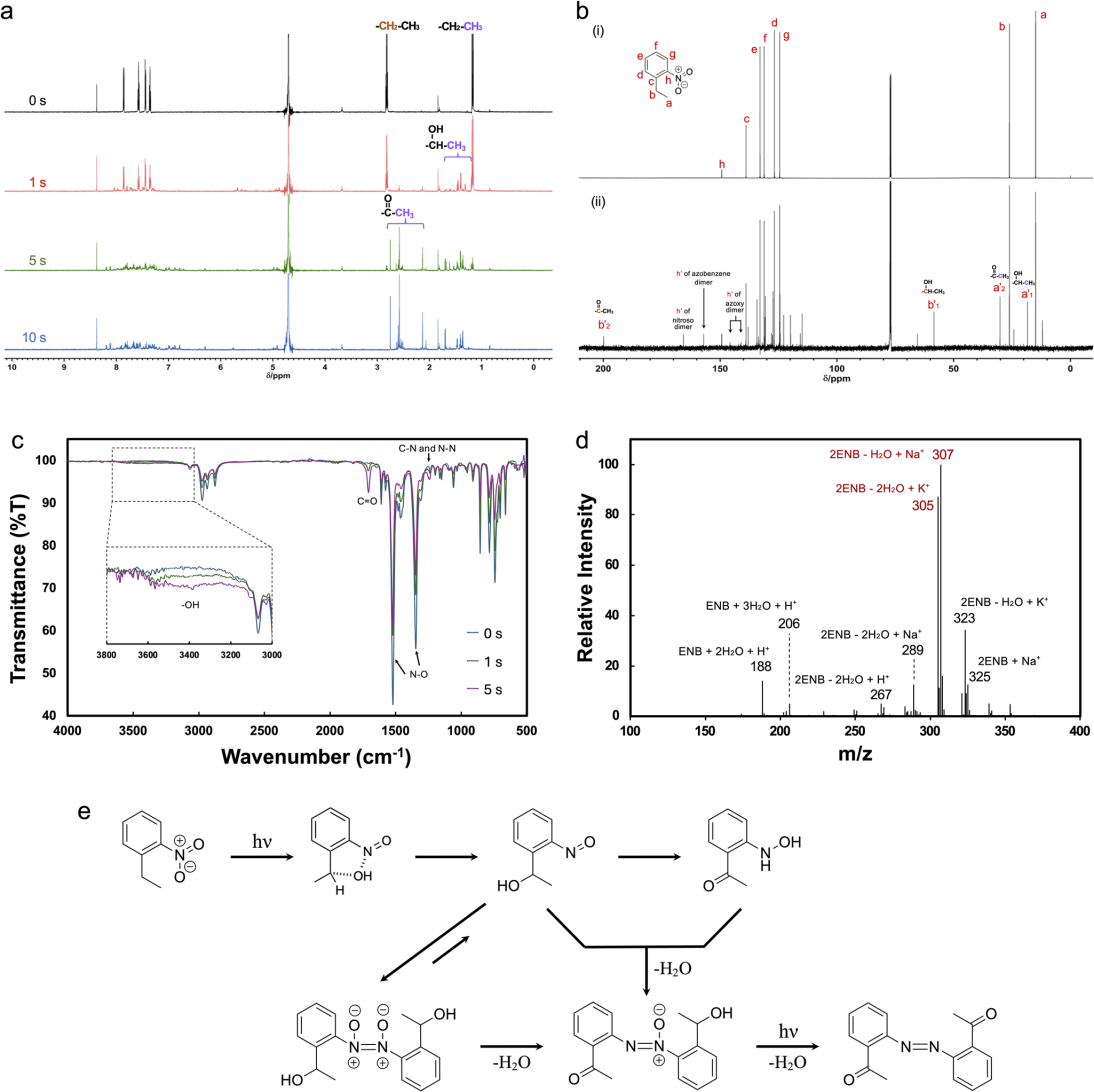
(a) Irradiation dose dependence of ^1^H NMR spectra of 2‐ethylnitrobenzene (ENB) in D_2_O. UV (365 nm, 240 mW cm^−^^2^) was irradiated for 0, 1, 5, and 10 s (600 MHz, 64 scans). (b) ^1^ ^3^C NMR spectra of ENB in CDCl_3_ before and after UV irradiation (365 nm, 240 mW cm^−^^2^, 60 s) (600 MHz, 512 scans). (c) Irradiation dose dependence of FT–IR (ATR) spectra of ENB in CDCl_3_ under UV irradiation (365 nm, 240 mW cm^−^^2^) for 0, 1, and 5 s. (d) Mass spectrum of ENB in ultrapure water after UV irradiation (365 nm, 240 mW cm^−^^2^, 30 s). (e) Schematic illustration of the photoreaction and photocrosslinking mechanism of ENB suggested from comprehensive spectroscopic analyses.

To further elucidate the photoreaction mechanism of the ENB moiety, light‑induced structural changes were analyzed with free ENB. First, temporal changes in the ^1^H NMR spectra of ENB dissolved in D_2_O were monitored under UV irradiation for defined time intervals. Figure S12 shows the correspondence between the ^1^H NMR peaks and the molecular structure of ENB prior to UV irradiation. As shown in the ^1^H NMR spectra in Figure 6a, the signals at approximately 1.2 and 2.9 ppm, corresponding to the methyl protons and the methylene protons of the ethyl group, respectively, gradually decreased and eventually disappeared upon photoirradiation. In contrast, after 1 s of irradiation, new signals appeared at around 1.4 ppm, and with further irradiation, signals appeared and increased in intensity around 2.6 ppm. The set of signals near 1.4 and 2.6 ppm can be attributed to methyl protons of α‑hydroxyethyl groups and to methyl protons of acetyl groups, respectively. Given that migration of an oxygen atom from the nitro group to an adjacent substituent has been reported in the photoreactions of many 1‐substituted aromatic nitro compounds [24, 25, 26, 27, 28], these changes in the ^1^H NMR spectra are suggested to reflect the oxidation of the ethyl group adjacent to the nitro group in ENB. The appearance of multiple signals in these regions is suggested to reflect the generation of diverse molecular species during the photoreaction, including reaction intermediates, several types of dimers and their isomers.

Figure 6b shows the change in the ^13^C NMR spectra of ENB dissolved in CDCl_3_ induced by UV irradiation. Before irradiation, signals of the α‑carbon and β‑carbon of the ethyl group were observed at approximately 26 and 15 ppm, respectively (Figure 6b (i)). After irradiation, signals appeared at around 58 and 19 ppm, which can be attributed to the α‑carbon and β‑carbon of an α‑ hydroxyethyl group. Signals also appeared at around 200 and 31 ppm, which can be attributed to the α‑carbon and β‑carbon of an acetyl group. These observations are consistent with the changes observed in the ^1^H‐NMR spectrum. Furthermore, we observed signals suggesting the photo‐dimerization of the molecules. Before irradiation, we observed a signal at approximately 149 ppm, which can be attributed to the aromatic carbon atom in the benzene ring to which the nitrogen atom is bonded. After irradiation, signals newly appeared at around 166 and 157 ppm, which can be attributed to a nitroso dimer and azobenzene, respectively. Small signals also appeared at around 141 and 146 ppm, suggesting the generation of azoxy dimers. Additionally, UV–vis spectroscopy of 0.1 wt.% ENBPVA solution revealed increased absorbance at ∼240 and ∼320 nm upon UV irradiation, as shown in Figure 2e. These spectral features are consistent with nitroso formation in other aromatic nitro compounds [48, 49].

The results of the Fourier transform infrared spectroscopy (FT‑IR) of ENB are shown in Figure 6c. Light irradiation decreased the absorbance bands at approximately 1520 and 1350 cm^−^^1^, which are attributed to the N─O asymmetric and symmetric stretching vibrations in the nitro group, respectively. In contrast, after irradiation, new bands appeared at around 3390 cm^−^^1^, 1705 cm^−^^1^, and 1240–1255 cm^−^^1^, and increased with increasing irradiation. The bands observed at approximately 3390 and 1705 cm^−^^1^ are attributed to O─H stretching vibrations and C═O stretching vibrations, respectively, suggesting the formation of α‑hydroxyethyl groups and acetyl groups. Furthermore, the bands observed in the 1240–1255 cm^−^^1^ region can be assigned to C─N or N─N stretching vibrations, suggesting the formation of hydroxylamine groups and azodioxide dimers. The bands derived from azoxy and azobenzene dimers are generally known to appear with weak intensity near 1530 and 1360 cm^−^^1^. In the spectra obtained in this study, however, these bands overlap with the N─O stretching vibrations of the nitro group and therefore are not observed as distinct new bands.

To directly observe photodimerized ENB, we carried out mass spectrometric analysis. Figure 6d shows the spectrum obtained after irradiating an aqueous ENB solution with light for 30 s. The prominent signals were observed at m/z 305 and 307, which can be assigned to [2ENB – 2H_2_O + K^+^] and to [2ENB – H_2_O + Na^+^], respectively. Together with other major signals that can also be attributed to the feasible derivatives of the ENB dimer, the formation of dimeric species was strongly suggested.

Taking into account all the results of the multifaceted analyses described above, together with previous reports on related photochemical systems, we propose the most plausible reaction pathway currently consistent with all experimental observations for the remarkable ENB‐mediated photocrosslinking phenomenon (Figure 6e). Although the individual elementary steps involved in this photochemical process have not been directly established, the proposed pathway is fully consistent with all spectroscopic analyses and experimentally observed photogelation behavior obtained in this study. In this proposed mechanism, UV irradiation of ENB induces intramolecular redox reactions, leading to hydroxylation at the α‐carbon of the adjacent ethyl group accompanied by the generation of a nitroso group, which is well known to undergo rapid dimerization. Unlike conventional photocrosslinking moieties such as Anth and Cum discussed above, the resulting nitroso groups are expected to maintain a dimerizable active state for a relatively long duration, thereby enabling the formation of effective intermolecular crosslinks. Although nitroso dimers are generally considered unstable, those derived from ENB are presumed to exhibit relatively high stability owing to the so‐called ortho effect. Subsequent redox processes and/or dehydration reactions are proposed to further transform the α‐carbon into a carbonyl group, resulting in the formation of azoxy dimers or related hydroxylamine derivatives that may contribute to the stabilization of the crosslinked network. This proposal is consistent with the observation that the ENBPVA hydrogel retained a mechanically stable shape even after reaching equilibrium swelling at a water content of 99.99 wt.%. In addition, further dehydration upon prolonged light irradiation of the azoxy dimers may lead to the formation of deeply colored azobenzene species, which is consistent with the coloration of the hydrogel observed in Figure 2d. Although further structural characterization employing more advanced analytical techniques will be necessary to directly elucidate the detailed photochemical processes involved, the present results collectively provide substantial support for the proposed reaction pathway and clearly demonstrate the exceptional ENB‐mediated photocrosslinking behavior observed in dilute aqueous polymer solutions.

### 2.4 Construction of 2D and 3D Cell Culture Systems Using ENBPVA Hydrogels

In addition to systems utilizing the aforementioned click reactions, photopolymerization systems based on macromonomers have been widely used to construct cell culture systems as described above [21, 22]. Nevertheless, there remains a demand for operation under conditions where radical generation is not feasible. In that regard, the photoinduced sol–gel transition of ENBPVA is distinctive in that gelation can be implemented with a single macromolecular component without the need for low‑molecular‑weight additives capable of penetrating cells. Furthermore, thanks to its radical‐free nature, this system is expected to be applicable to a wide range of bioapplications. In cell viability assays of ENBPVA, high cell viability was maintained even 24 h after treatment with ENBPVA (Figure S13).

**FIGURE 7:**
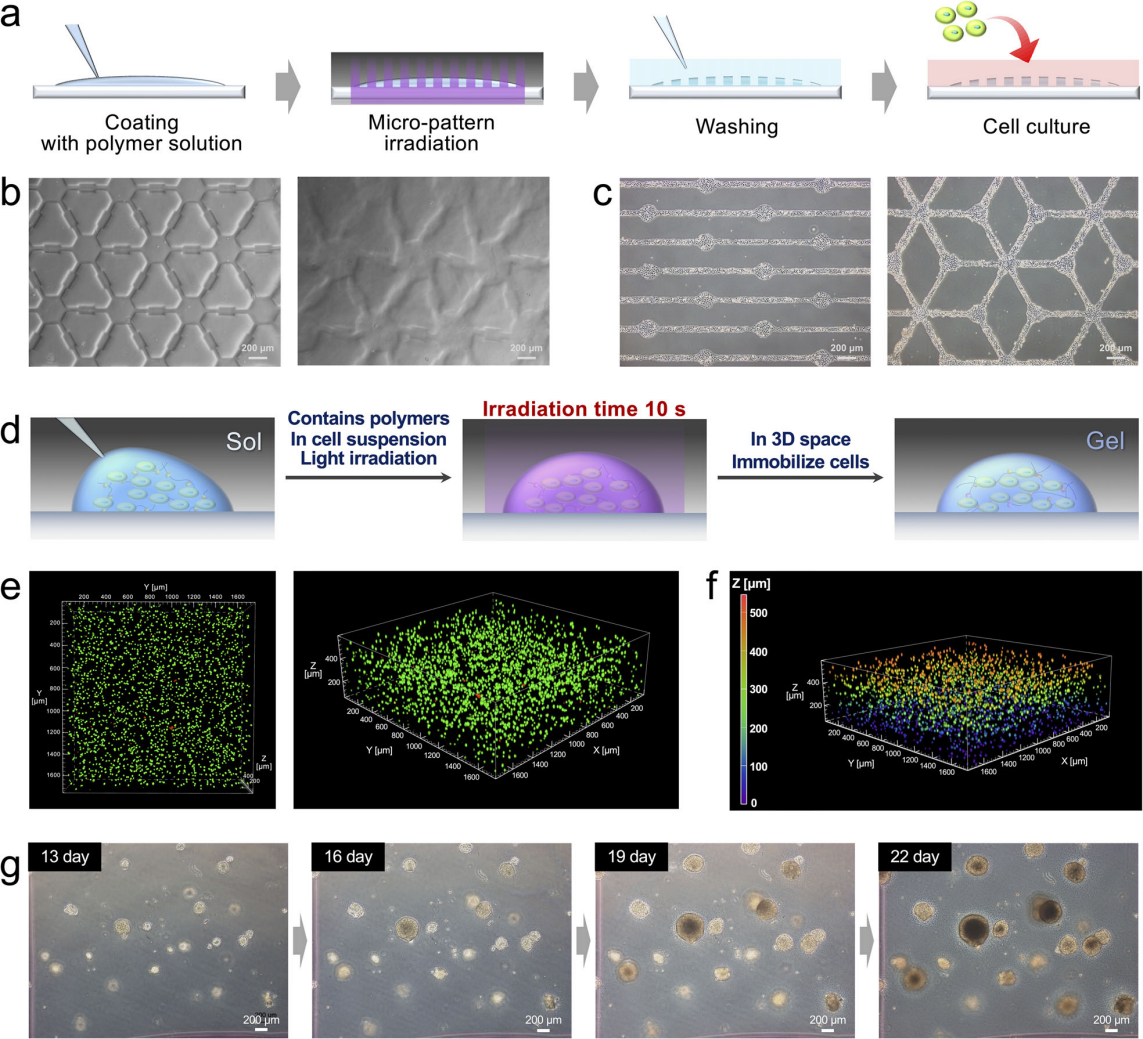
(a) Schematic illustration of a cell patterning process involving a series of steps: ENBPVA solution was coated onto a polystyrene dish, followed by UV irradiation through a micropatterned photomask and subsequent washing, resulting in a patterned hydrogel on a cell culture substrate. (b) Phase‐contrast microscopy images of the resulting hydrogel pattern before (left) and after swelling (right). (c) Phase‐contrast microscopy images of the cell pattern one day after seeding HeLa cells onto the hydrogel pattern: a combination of lines and dots (left), and a hexagonal pattern (right). (d) Schematic illustration showing the construction process of an in‐gel cell culture system. Cells are immobilized immediately in 3D space by UV irradiation of an ENBPVA‐containing cell suspension. (e) After one day of in‐gel culture, live cells were stained with Calcein AM (green) and dead cells with Ethidium Homodimer‐1 (red). Z‐stack fluorescence images were obtained using confocal laser scanning microscopy and reconstructed into 3D fluorescence images. (f) 3D fluorescence image obtained after one day of in‐gel culture, with pseudo‐color representation of cell position along the Z‐axis. (g) Phase‐contrast microscopy images acquired over time of HeLa cells cultured in ENBPVA hydrogels. Spheroid formation was promoted by long‐term periodic incubation (up to 22 days) in the 3D environment of the hydrogel.

We first investigated the fabrication of microstructures using precise light irradiation (Figure 7a). A PBS solution containing 1 wt.% ENBPVA was applied as a thin (sub‐millimeter) layer on a polystyrene surface, followed by micropatterned UV exposure. After washing with water and drying, a micrograph revealed that the crosslinked polymer layer remained only in the irradiated regions (Figure 7b, left), demonstrating the feasibility of microfabrication via photolithography. Furthermore, the crosslinked layer could be rehydrated by applying water, showing reversible swelling behavior (Figure 7b, right). When scaffold‐dependent cells were seeded onto the surface, they adhered only to the hydrogel‐free areas and proliferated within these regions, resulting in patterned cell cultures (Figure 7c).

To further explore the bioapplications of ENBPVA, we investigated the construction of an in‐gel culture system based on photo‐induced gelation of an ENBA‐containing cell suspension (Figure 7d). A dispersion of HeLa cells in culture medium containing 1 wt.% ENBPVA was poured on a 35‐mm‐diameter polystyrene culture dish and irradiated with UV light before cell sedimentation. Figure 7e presents a 3D image generated from Z‐stack images obtained by confocal laser microscopy after one day of in‐gel cell culture. The image clearly shows that calcein‐stained live cells were immobilized within the 3D network of the hydrogel. Figure 7f shows a 3D image in which cells are color‐coded along the Z‐axis according to their vertical position, demonstrating uniform spatial distribution of the cells throughout the hydrogel matrix. This demonstrates that the medium can be converted into a hydrogel and the cells can be immobilized in a defined 3D space prior to the settling of cells or cell aggregates, without sudden temperature changes, addition of active substances, or mechanical agitation. After adding 1.5 mL of culture medium, the hydrogel swelled shortly after but remained structurally stable for over a month. Cells immobilized three‐dimensionally within the hydrogel remained viable and formed spheroids up to 500 µm in diameter after one month of culture (Figure 7g). These findings indicate that polymer incorporation and photocrosslinking did not compromise cell viability. In the hydrogel composed of >99% culture medium, it was suggested that oxygen and nutrients diffused freely through the polymer network, as in aqueous solutions [50]. These results demonstrate the on‐demand construction of a 3D culture system that does not rely on substrate adhesion, yet supports long‐term medium exchange while maintaining cells in stable spatial distribution.

These results suggest that ENBPVA is expected to be a platform for minimally invasive in vitro bioapplications such as the development of new cell culture systems. On the other hand, we should exercise caution regarding the in vivo use of the polymer containing ENB, an aromatic nitro compound, even if its content in the resulting hydrogel is extremely small and it is bound to the polymer network.

## 3 Conclusion

We have demonstrated a remarkable photocrosslinking system newly discovered in this study on ENB‐functionalized PVA. At just 0.6 mol% functionalization, a polymer solution with a concentration of 1 wt.% underwent a pronounced sol–gel transition to a hydrogel exhibiting both flexibility and high compressive fracture resistance upon a very short light irradiation (0.5 s), demonstrating that ENB is a highly efficient and compact photo‐crosslinkable motif of a new class. The hydrogel swelled in water, retaining sufficient mechanical strength to be handled manually even after its water content reached 99.99 wt.%. Based on multiple analyses, we propose a plausible mechanism for this remarkable ENB‐mediated photocrosslinking phenomenon, which begins with the generation of dimerizable species with relatively long‐lived activity. In the in‐gel culture system established using this approach, in situ photogelation was found not to compromise the viability of the embedded cells, and scaffold‐dependent cells formed large spheroids, demonstrating the cytocompatibility of this system.

This highly efficient photo‐induced sol–gel transition system offers not only cytocompatibility but also compatibility with optical engineering technologies. In particular, its ability to instantaneously induce gelation at the centimeter scale upon light irradiation holds significant potential for applications in 3D printing technology. Furthermore, when combined with our previously reported photo‐responsive polymer materials that undergo light‐triggered dissolution, this photocrosslinking system is expected to provide a versatile platform for on‐demand control of cell culture within soft, highly hydrated biological environments.

## 4 Experimental Section/Methods

### 4.1 Materials

Poly(vinyl alcohol) (PVA, Mowiol 56–98, #10851, Sigma–Aldrich Co.) with an average molecular weight of approximately 195 000 g/mol and a degree of hydrolysis of 98.0–98.8% was used in this study. 4‐Ethyl‐3‐nitrobenzaldehyde (ENBA, #634085, Sigma–Aldrich Co.), glyoxylic acid monohydrate (GoAc, #079‐06812, FUJIFILM Wako Pure Chemical Corp.), 9‐Anthracenecarboxaldehyde (#A0779, Tokyo Chemical Industry Co., Ltd.), and 6‐Formaldehydecoumarin (#510‐44131, FUJIFILM Wako Pure Chemical Corp.) were used as formylating agents to introduce photocrosslinking motifs and carboxyl groups into the PVA backbone. Non‐treated polystyrene petri dishes (35 mm diameter, #1000‐035, AGC TECHNO GLASS Co., Ltd.) and tissue culture polystyrene (TCPS) petri dishes (35 mm diameter, #3000‐035, AGC TECHNO GLASS Co., Ltd.) were used as the base substrates for cell culture experiments. HepG2 cells were obtained from the RIKEN Bioresource Center (Tsukuba, Ibaraki, Japan). For HepG2 cell culture, Dulbecco's Modified Eagle Medium with high glucose (D‐MEM, #045‐30285, FUJIFILM Wako Pure Chemical Corp.) supplemented with 10% fetal bovine serum (FBS, #SH30396.03, HyClone), penicillin–streptomycin (#168‐23191, FUJIFILM Wako Pure Chemical Corp.), and MEM non‐essential amino acids (#139‐15651, FUJIFILM Wako Pure Chemical Corp.) was used. Dulbecco's phosphate‐buffered saline without calcium and magnesium (D‐PBS (–), #045‐29795, FUJIFILM Wako Pure Chemical Corp.)was used to prepare ENBPVA solutions for in‐gel culture experiments. Buffer solution standard (Phosphate pH standard equimolar solution) pH 6.86 (#025‐03195, FUJIFILM Wako Pure Chemical Corp.) was used to prepare ENBPVA solutions for dynamic light scattering (DLS) measurement. 2,2,6,6‑tetramethylpiperidin‑1‑oxyl (TEMPO, #T1560, Tokyo Chemical Industry Co., Ltd.). Chloroform‐d (CDCl_3_, # 039–25113, FUJIFILM Wako Pure Chemical Corp.) and Deuterium Oxide (D_2_O, # 047–34243, FUJIFILM Wako Pure Chemical Corp.).

### 4.2 Apparatus

UV irradiation was performed using LED light sources (SOLIS‐365C and SOLIS‐385C, Thorlabs) at wavelengths of 365 nm and 385 nm, respectively. NMR spectra were acquired on a 600 MHz NMR spectrometer (AVANCE III HD 600, Bruker Corp.). Absorption spectra and Transmittance were recorded using a UV–vis spectrophotometer (V‐750, JASCO Co.). FT–IR spectra were recorded using an FT–IR spectrometer (VIR‐100, JASCO Co.). Mass spectra were obtained using a mass spectrometer (LCMS‐2020, Shimadzu Co.). Adhesion strength measurements and compression tests were performed using Material Testing Instruments (MCT‐2150W, A&D Co., Ltd.). Hydrodynamic diameter of ENBPVA aggregation was measured using a DLS apparatus with a 633 nm He‐Ne laser (Zetasizer Nano‐ZS, Malvern Instruments Ltd.). A spin coater (ASS‐301, Able Co., Ltd.) was used for coating the polymer solutions. Microscopic images of cultured cells were acquired using a cooled CCD camera system (VB‐7000, Keyence Co.) mounted on an inverted research microscope (IX70, Olympus Co.) equipped with a 4× objective lens (Plan Apo 4×, Olympus Co.). 3D and fluorescence imaging was conducted using a confocal laser scanning microscope (AX with NSPARC, NIKON Co.). Cell culture was carried out in a CO_2_ incubator (SMA‐165DRS, ASTEC Co., Ltd.).

### 4.3 Synthesis of ENBPVA, AnthPVA, and CumPVA, and Preparation of Their Aqueous Solutions

A typical synthesis procedure for ENBPVA is described as follows. Poly(vinyl alcohol) (PVA) was first dissolved in DMSO by stirring to obtain a 7.7 wt.% solution. To this solution, 0.037 g of 4‐ethyl‐3‐nitrobenzaldehyde (ENBA), 0.46 g of glyoxylic acid monohydrate, and 0.092 g of urea were added and stirred until homogeneous. The acetalization reaction was initiated by adding a mixture of 0.53 g of 1 N HCl and 0.14 g of DMSO to the reaction mixture, followed by continued stirring. The reaction was carried out at 75 °C for 2 days. After completion, the reaction was quenched by the addition of 0.64 g of 1 M NaOH solution. Subsequently, 30 mL of ethyl acetate was added to precipitate the polymer. The precipitate was washed with ethanol and dried at 80 °C, yielding 0.82 g of ENBPVA as a solid. To prepare aqueous solutions, the obtained ENBPVA was dissolved in 0.1 N sodium hydroxide solution with stirring and heating, affording solutions at concentrations ranging from 1 to 3 wt.%. For experiments involving cell manipulation, a 2 wt.% ENBPVA solution was also prepared by dissolving the polymer in Dulbecco's phosphate‐buffered saline (D‐PBS, pH 7.4). AnthPVA and CumPVA were synthesized using 9‐anthracenecarboxaldehyde and 6‐formylcoumarin, respectively, following the same procedure as that used for ENBPVA. Their aqueous solutions were also prepared at the concentrations used for each measurement, in the same manner as ENBPVA.

### 4.4 Characterization of ENBPVA

The successful synthesis of ENBPVA was confirmed by proton nuclear magnetic resonance (^1^H NMR) spectroscopy. The spectra were acquired using a Bruker AVANCE III HD 600 MHz spectrometer, with D_2_O as the solvent and the internal standard. For UV–visible (UV–vis) spectroscopy, ENBPVA was dissolved in water at a concentration of 0.1 wt.%. The solution was irradiated with UV light at 365 nm (240 mW cm^−^^2^) for various durations using an LED light source.

### 4.5 Preparation of ENBPVA Hydrogels

ENBPVA hydrogels were prepared by depositing 20  µL of a 2 wt.% ENBPVA aqueous solution onto a silicone sheet placed on top of a UV light source, followed by exposure to 365 nm UV light for 5 s. As shown in Figure 2d, a bear‐shaped ENBPVA hydrogel with a thickness of 1 cm was fabricated by pouring a 1.5 wt.% ENBPVA aqueous solution into a silicone mold shaped like a bear and irradiating it with 385 nm UV light for a defined period. To compare the gelation behavior of ENBPVA, AnthPVA, and CumPVA, aqueous solutions were prepared at six concentrations ranging from 0.5 to 3 wt.%. Hydrogel formation was assessed at nine irradiation times (0.5–120 s) based on the response observed upon contact with a wipe (Figure S5).

### 4.6 Swelling Behavior of ENBPVA Hydrogels

ENBPVA hydrogels were prepared by depositing 50  µL of a 2 wt.% ENBPVA aqueous solution onto a silicone sheet placed over a UV light source, followed by irradiation at 365 nm for 5 s. The resulting hydrogels were transferred to Petri dishes and immersed in ultrapure water for swelling. In Figure 2f, the external solvent (ultrapure water) was replaced six times over a 24 h period. In contrast, in Figure 2g, the external solvent (ultrapure water) was not replaced. At predetermined time intervals, either the weight (*W*) or diameter (*D*) of the hydrogels was measured, and the swelling ratio was calculated using Equation (1).

(1) $$\mathrm{Swelling}\;\mathrm{ratio}=\frac{W_{\mathrm{after}\;\mathrm{swelling}}}{W_{\mathrm{as}\;\mathrm{prepared}}}={\left(\frac{D_{\mathrm{after}\;\mathrm{swelling}}}{D_{\mathrm{as}\;\mathrm{prepared}}}\right)}^{3}$$

### 4.7 Compression Testing of ENBPVA Hydrogels

Cylindrical ENBPVA hydrogels (3 mm in diameter, 2 mm in height) were prepared by pouring ENBPVA aqueous solutions of defined concentrations into silicone molds, followed by UV irradiation at 365 nm for specified durations. Compression tests were conducted using a universal testing machine under a maximum load of 150 N and a maximum strain of 2 mm. Stress–strain curves were recorded, and the maximum compressive stress and strain were determined. To assess the effect of UV irradiation time, 1.5 wt.% ENBPVA solution was used, while the concentration dependence was evaluated at a fixed irradiation time of 10 s. To compare the compressive stress of AnthPVA and CumPVA with that of ENBPVA, compression tests were performed using hydrogels prepared under the lowest polymer concentration and shortest irradiation time at which stable cylindrical specimens (2.5 mm in diameter and 2 mm in height) could be formed. AnthPVA hydrogels were prepared at a polymer concentration of 3 wt.% with 30 s of irradiation, whereas CumPVA hydrogels were prepared at 10 wt.% with 300 s of irradiation. To obtain swollen hydrogels, ENBPVA hydrogels prepared by irradiating 2 wt.% ENBPVA solution with 365 nm UV light for 10 s were immersed in ultrapure water for predetermined periods. The swollen samples were subsequently subjected to compression testing under the same conditions described above. The modulus of elasticity of the hydrogel was determined from the stress‐strain curve obtained in a compression test using Equation (2), where σ is the compressive stress, *G* is the modulus of elasticity, and α is the ratio of the hydrogel thickness before and after compression. The plot of σ versus (α – α^−2^) showed a linear relationship, and the slope of this line gave the value of *G*. The effective crosslink density ν*_eff_* of the hydrogel was calculated using Equation (3) [30].

(2) $$\sigma =G\left(\alpha -\;\alpha^{-2}\right)$$

(3) $$G\;\approx \mathrm{RT}\nu_{\mathrm{eff}}$$

To evaluate the tensile strength of the ENBPVA hydrogel where no significant deformation occurs, two cylindrical acrylic resin (PMMA) rods (30 mm in diameter, 50 mm in height) were joined by applying 10  µL of a 3 wt.% ENBPVA aqueous solution between their end faces, followed by UV irradiation at 365 nm for 40 s to form a hydrogel thin layer. The bonded pieces of rods were suspended vertically via a rope connected to a tensile testing machine, and a tensile test was performed with a maximum load of 200 N to determine the tensile strength of the hydrogel.

### 4.8 Hydrodynamic diameters of polymer cross‐linked bodies measured by DLS

A 0.2% ENBPVA solution in a phosphate buffer solution (PBS) with a pH of 6.86 was prepared for DLS measurements to appropriately evaluate the hydrodynamic size of charged polymer clusters under well‐shielded conditions. Two different conditions of irradiation intensity were set to 250 and 4 mW cm^−^^2^. Measurement was carried out three times at the irradiation times of 5, 10, and 20 s at an intensity of 250 mW cm^−^^2^, and at 5, 10, 15, and 20 min at an intensity of 4 mW cm^−^^2^.

### 4.9 Evaluation of Photocrosslinking Mechanism of ENB and ENBPVA

First, changes in the physical properties were examined by irradiating a mixed aqueous solution of polyvinyl alcohol (PVA) and 4‐ethyl‐3‐nitrobenzoic acid (ENBAc), a water‐soluble small molecule containing an ENB group, with 365 nm UV light for 30 s. Subsequently, three types of solutions were prepared: 1.5 wt.% ENBPVA alone, 1.5 wt.% ENBPVA with added ENBAc, and 1.5 wt.% ENBPVA with added benzoic acid (as a control). The amounts of ENB groups in 1.5 wt.% ENBPVA and the added ENBAc or benzoic acid were adjusted to be equimolar. After 30 s of UV irradiation at 365 nm, gelation of each sample was evaluated by its adsorption behavior on a paper wiper. To confirm that gelation does not proceed via radical species, TEMPO (5 wt.%) was added to a 2 wt.% ENBPVA aqueous solution. After thorough stirring, hydrogel formation was observed upon UV irradiation (365 nm, 240 mW cm^−^^2^, 10 s). To evaluate structural changes, ^1^H NMR spectroscopy was used to monitor the spectral changes of ENB under UV irradiation (365 nm, 240 mW cm^−^^2^) for 0, 1, 5, and 10 s. Measurements were performed at an ENB concentration of 0.01 wt.% in D_2_O with 64 scans. ^1^ ^3^C NMR spectroscopy was used to investigate the spectral changes of ENB before and after 60 s of UV irradiation (365 nm, 240 mW cm^−^^2^). Measurements were performed at an ENB concentration of 0.1 wt.% in CDCl_3_ with 512 scans. FT–IR spectra were recorded in ATR mode using an FT–IR spectrometer with 128 scans. To evaluate structural changes in ENB, spectra were collected after stepwise UV irradiation (365 nm, 240 mW cm^−^^2^) for 0, 1, 3, and 5 s using a 0.5 wt.% ENB solution in CDCl_3_. Mass spectra were acquired in positive ion mode using an electrospray ionization (ESI) mass spectrometer. Samples were prepared at an ENB concentration of 0.1 mM in ultrapure water and analyzed after UV irradiation (365 nm, 240 mW cm^−^^2^, 30 s).

### 4.10 Cytotoxicity Testing of ENBPVA

One day after seeding mouse fibroblast 3T3 cells into 96‐well plates, the culture medium was replaced with fresh medium containing a predetermined concentration of ENBPVA and incubated for an additional day in a CO_2_ incubator. Cell viability was then assessed using the WST‐8 assay with the Cell Counting Kit‐8 (DOJINDO LAB). The viability of cells cultured in medium without ENBPVA was defined as 100%.

### 4.11 Preparation of ENBPVA Pattern Hydrogels and Cell Pattern Culture

A 0.5% ENBPVA solution in hexafluoroisopropanol containing 20% water was loaded into the center of a TCPS petri dish at 8 µL and spin‐coated at 1200 rpm. Immediately thereafter, UV light (365 nm, 190 mW cm^−^^2^) for 5 s through a predetermined photomask to induce gelation locally. The surface was then washed with water to remove uncrosslinked ENBPVA. HeLa cells dispersed in culture medium were seeded onto the thus patterned dish surface, incubated in a CO_2_ incubator for one day, and observed under a microscope.

### 4.12 Construction of HepG2 In‐Gel Culture System

A 2 wt.% ENBPVA solution in D‐PBS(‐) was added to a culture medium (containing 10% FBS) in which HepG2 cells were suspended, and the mixture was gently stirred. Then, 70  µL of the cell suspension was cast onto the bottom of a 35 mm dish and immediately irradiated with UV light (365 nm, 250 mW cm^−^^2^) for 15 s to induce gelation. Culture medium was then added around the hydrogel, and the culture was continued for 22 days in a CO_2_ incubator with periodic medium changes and microscopic observations. After one day of in‐gel culture, live cells were stained with Calcein‐AM and dead cells with Ethidium Homodimer‐1. Z‐stack fluorescence images were acquired using confocal laser microscopy and reconstructed into 3D fluorescence images.

## Author Contributions

**Masaaki Okihara**: Writing – original draft, Writing – review and editing, investigation, validation, formal analysis, conceptualization, methodology. **Toshiyuki Takagi**: investigation, validation, methodology. **Kana Morishita**: investigation. **Ayaka Tomoda**: investigation. **Kimio Sumaru**: conceptualization, methodology, supervision, funding acquisition, writing – review and editing, investigation, validation.

## Funding

This study was supported in part by the KAKENHI Grant‐in‐Aid for JSPS Fellows (JP23KJ2110) and that for Scientific Research B (19H02578) from the Japan Society for the Promotion of Science (JSPS). This study was in part supported by Adaptable and Seamless Technology transfer Program through Target‐driven R&D (A‐STEP) from Japan Science and Technology Agency (JST) Japan Grant Number JPMJTR25UD.

## Conflicts of Interest

The authors declare no conflict of interest.

## Supporting information




**Supporting File 1**: advs77119‐sup‐0001‐SuppMat.pdf.

**Supporting File 2**: advs77119‐sup‐0002‐S1.mp4.

**Supporting File 3**: advs77119‐sup‐0003‐S2.mp4.

**Supporting File 4**: advs77119‐sup‐0004‐S3.mp4.

**Supporting File 5**: advs77119‐sup‐0005‐S4.mp4.

**Supporting File 6**: advs77119‐sup‐0006‐S5.mp4.

**Supporting File 7**: advs77119‐sup‐0007‐S6.mp4.

**Supporting File 8**: advs77119‐sup‐0008‐S7.mp4.

## Data Availability

The data that supports the findings of this study are available in the supplementary material of this article.

## References

[1] S. L. Scinto , D. A. Bilodeau , R. Hincapie , et al., “Bioorthogonal Chemistry,” Nature Reviews Methods Primers 1, no. 1 (2021): 30, 10.1038/s43586-021-00028-z. PMC8469592 34585143

[2] R. E. Bird , S. A. Lemmel , X. Yu , and Q. A. Zhou , “Bioorthogonal Chemistry and Its Applications,” Bioconjugate Chemistry 32, no. 12 (2021): 2457–2479, 10.1021/acs.bioconjchem.1c00461. 34846126

[3] H. Sun , C. P. Kabb , M. B. Sims , and B. S. Sumerlin , “Architecture‐Transformable Polymers: Reshaping the Future of Stimuli‐Responsive Polymers,” Progress in Polymer Science 89 (2019): 61–75, 10.1016/j.progpolymsci.2018.09.006.

[4] E. S. Gil and S. M. Hudson , “Stimuli‐Reponsive Polymers and Their Bioconjugates,” Progress in Polymer Science 29, no. 12 (2004): 1173–1222, 10.1016/j.progpolymsci.2004.08.003.

[5] L. Li , J. M. Scheiger , and P. A. Levkin , “Design and Applications of Photoresponsive Hydrogels,” Advanced Materials 31, no. 26 (2003): 1807333, 10.1002/adma.201807333. PMC9285504 30848524

[6] G. Kaur , P. Johnston , and K. Saito , “Photo‐Reversible Dimerisation Reactions and Their Applications in Polymeric Systems,” Polymer Chemistry 5, no. 7 (2014): 2171–2186, 10.1039/C3PY01234D.

[7] L. Klouda and A. G. Mikos , “Thermoresponsive Hydrogels in Biomedical Applications,” European Journal of Pharmaceutics and Biopharmaceutics 68, no. 1 (2008): 34–45, 10.1016/j.ejpb.2007.02.025. 17881200 PMC3163097

[8] I. S. Protsak and Y. M. Morozov , “Fundamentals and Advances in Stimuli‐Responsive Hydrogels and Their Applications: A Review,” Gels 11, no. 1 (2025): 30, 10.3390/gels11010030. 39852001 PMC11765116

[9] J. Hoque , N. Sangaj , and S. Varghese , “Stimuli‐Responsive Supramolecular Hydrogels and Their Applications in Regenerative Medicine,” Macromolecular Bioscience 19, no. 1 (2018): 1800259, 10.1002/mabi.201800259. PMC6333493 30295012

[10] H. M. El‐Husseiny , E. A. Mady , L. Hamabe , et al., “Smart/Stimuli‐Responsive Hydrogels: Cutting‐Edge Platforms for Tissue Engineering and other Biomedical Applications,” Materials Today Bio 13 (2022): 100186, 10.1016/j.mtbio.2021.100186. PMC8669385 34917924

[11] R. Wakabayashi , W. Ramadhan , K. Moriyama , M. Goto , and N. Kamiya , “Poly(ethylene glycol)‐Based Biofunctional Hydrogels Mediated by Peroxidase‐Catalyzed Cross‐Linking Reactions,” Polymer Journal 52, no. 8 (2020): 899–911, 10.1038/s41428-020-0344-7.

[12] A. A. H. Bukhari , N. H. Elsayed , and M. Monier , “Development and Characterization of Photo‐Responsive Cinnamoly Modified Alginate,” Carbohydrate Polymers 260 (2021): 117771, 10.1016/j.carbpol.2021.117771. 33712129

[13] M. Okihara , K. Okuma , A. Kawamura , and T. Miyata , “Photoresponsive Gelation of Four‐Armed Poly(Ethylene Glycol) with Photodimerizable Groups,” Gels 8, no. 3 (2022): 183, 10.3390/gels8030183. 35323296 PMC8950757

[14] L. A. Wells , M. A. Brook , and H. Sheardown , “Generic, Anthracene‐Based Hydrogel Crosslinkers for Photo‐Controllable Drug Delivery,” Macromolecular Bioscience 11, no. 7 (2011): 988–998, 10.1002/mabi.201100001. 21604375

[15] Y. Zheng , M. Micic , S. V. Mello , et al., “PEG‐Based Hydrogel Synthesis via the Photodimerization of Anthracene Groups,” Macromolecules 35, no. 13 (2002): 5228–5234, 10.1021/ma012263z.

[16] M. Okihara , A. Matsuda , A. Kawamura , and T. Miyata , “Design of dual Stimuli‐Responsive Gels With Physical And Chemical Properties That Vary In Response To Light And Temperature And Cell Behavior On Their Surfaces,” Polymer Journal 56, no. 3 (2024): 193–204, 10.1038/s41428-023-00865-7.

[17] R. Beninatto , C. Barbera , O. De Lucchi , et al., “Photocrosslinked hydrogels From Coumarin Derivatives Of Hyaluronic Acid For Tissue Engineering Applications,” Materials Science and Engineering: C 96 (2019): 625–634, 10.1016/j.msec.2018.11.052. 30606574

[18] T. A. Neuendorf , N. Weigel , M. Vigogne , and J. Thiele , “Additive Soft Matter Design by UV‐Induced Polymer Hydrogel Inter‐Crosslinking,” Gels 8, no. 2 (2022): 117, 10.3390/gels8020117. 35200499 PMC8871859

[19] K. Ichimura and S. Watanabe , “Preparation and Characteristics of Photocross‐Linkable Poly(Vinyl Alcohol),” Journal of Polymer Science: Polymer Chemistry Edition 20, no. 6 (1982): 1419–1432, 10.1002/pol.1982.170200605.

[20] K. Yang and M. Zeng , “Multiresponsive Hydrogel Based on Polyacrylamide Functionalized With Thymine Derivatives,” New Journal of Chemistry 37, no. 4 (2013): 920–926, 10.1039/C3NJ41013G.

[21] C. Yu , J. Schimelman , P. Wang , et al., “Photopolymerizable Biomaterials and Light‐Based 3D Printing Strategies for Biomedical Applications,” Chemical Reviews 120, no. 19 (2020): 10695–10743, 10.1021/acs.chemrev.9b00810. 32323975 PMC7572843

[22] Y. Hu , Z. Luo , and Y. Bao , “Trends in Photopolymerization 3D Printing for Advanced Drug Delivery Applications,” Biomacromolecules 26, no. 1 (2025): 85–117, 10.1021/acs.biomac.4c01004. 39625843 PMC11733939

[23] K. Sumaru , T. Takagi , K. Morishita , and T. Kanamori , “Photoresponsive Aqueous Dissolution of Poly(N‐Isopropylacrylamide) Functionalized with o‐Nitrobenzaldehyde through Phase Transition,” Biomacromolecules 19, no. 7 (2018): 2913–2922, 10.1021/acs.biomac.8b00470. 29641886

[24] P. Wan and K. Yates , “Photoredox Chemistry of Nitrobenzyl Alcohols in Aqueous Solution. Acid and Base Catalysis of Reaction,” Canadian Journal of Chemistry 64, no. 10 (1986): 2076–2086, 10.1139/v86-343.

[25] D. A. Fletcher , B. G. Gowenlock , K. G. Orrell , et al., “4‐Iodonitrosobenzene. Structural and Spectroscopic Studies of the Monomeric Solid and of Previously Unreported Dimers,” Journal of the Chemical Society, Perkin Transactions 2 2, no. 2 (1996): 191–197, 10.1039/P29960000191.

[26] B. G. Gowenlock , M. J. Maidment , K. G. Orrell , et al., “The Solid‐ and Solution‐State Structures of 2‐Nitrosopyridine and its 3‐ and 4‐Methyl Derivatives,” Journal of the Chemical Society, Perkin Transactions 2, no. 11 (2000): 2280–2286, 10.1039/B004270F.

[27] T. Liu , B. Bao , Y. Li , Q. Lin , and L. Zhu , “Photo‐Responsive Polymers Based on ο‐Nitrobenzyl Derivatives: From Structural Design to Applications,” Progress in Polymer Science 146 (2023): 101741, 10.1016/j.progpolymsci.2023.101741.

[28] H. Zhao , E. S. Sterner , E. B. Coughlin , and P. Theato , “o ‐Nitrobenzyl Alcohol Derivatives: Opportunities in Polymer and Materials Science,” Macromolecules 45, no. 4 (2012): 1723–1736, 10.1021/ma201924h.

[29] Q. Wang , J. L. Mynar , M. Yoshida , et al., “High‐water‐Content Mouldable Hydrogels by Mixing Clay and a Dendritic Molecular Binder,” Nature 463, no. 7279 (2010): 339–343, 10.1038/nature08693. 20090750

[30] P. H. J. Kouwer , M. Koepf , V. A. A. Le Sage , et al., “Responsive Biomimetic Networks from Polyisocyanopeptide Hydrogels,” Nature 493, no. 7434 (2013): 651–655, 10.1038/nature11839. 23354048

[31] W. Xue , M. B. Huglin , and T. G. J. Jones , “Swelling and Network Parameters of Crosslinked Thermoreversible Hydrogels of Poly(N‐Ethylacrylamide),” European Polymer Journal 41, no. 2 (2005): 239–248, 10.1016/j.eurpolymj.2004.10.005.

[32] C. Norioka , Y. Inamoto , C. Hajime , A. Kawamura , and T. Miyata , “A Universal Method to Easily Design Tough and Stretchable Hydrogels,” NPG Asia Materials 13, no. 1 (2021): 34, 10.1038/s41427-021-00302-2.

[33] A. Thangprasert , C. Tansakul , and N. Thuaksubun , “Mimicked Hybrid Hydrogel Based on Gelatin/PVA for Tissue Engineering in Subchondral Bone Interface for Osteoarthritis Surgery,” Materials & Design 183 (2019): 108113, 10.1016/j.matdes.2019.108113.

[34] C. M. Tierney , M. G. Haugh , J. Liedl , F. Mulcahy , B. Hayes , and F. J. O'Brien , “The Effects Of Collagen Concentration And Crosslink Density On The Biological, Structural And Mechanical Properties Of Collagen‐Gag Scaffolds For Bone Tissue Engineering,” Journal of the Mechanical Behavior of Biomedical Materials 2, no. 2 (2009): 202–209, 10.1016/j.jmbbm.2008.08.007. 19627824

[35] D. Lu , Z. Zeng , Z. Geng , et al., “Macroporous Methacrylated Hyaluronic Acid Hydrogel with Different Pore Sizes for In Vitro and In Vivo Evaluation of Vascularization,” Biomedical Materials 17, no. 2 (2022): 025006, 10.1088/1748-605X/ac494b. 34996058

[36] U. G. T. M. Sampath , Y. C. Ching , C. H. Chuah , R. Singh , and P. C. Lin , “Preparation and Characterization Of Nanocellulose Reinforced Semi‐Interpenetrating Polymer Network Of Chitosan Hydrogel,” Cellulose 24, no. 5 (2017): 2215–2228, 10.1007/s10570-017-1251-8.

[37] V. Normand , D. L. Lootens , E. Amici , K. P. Plucknett , and P. Aymard , “New Insight Into Agarose Gel Mechanical Properties,” Biomacromolecules 1, no. 4 (2000): 730–738, 10.1021/bm005583j. 11710204

[38] T. Boontheekul , H. J. Kong , and D. J. Mooney , “Controlling Alginate Gel Degradation Utilizing Partial Oxidation And Bimodal Molecular Weight Distribution,” Biomaterials 26, no. 15 (2005): 2455–2465, 10.1016/j.biomaterials.2004.06.044. 15585248

[39] T. L. Wang , Z. F. Zhou , J. F. Liu , X. D. Hou , Z. Zhou , et al., “Donut‐Like MOFs of Copper/Nicotinic Acid And Composite Hydrogels with Superior Bioactivity for rh‐bFGF Delivering and Skin Wound Healing,” Journal of Nanobiotechnology 19, no. 1 (2021): 275, 10.1186/s12951-021-01014-z. 34503490 PMC8427876

[40] S. J. Bryant , T. T. Chowdhury , D. A. Lee , D. L. Bader , and K. S. Anseth , “Crosslinking Density Influences Chondrocyte Metabolism In Dynamically Loaded Photocrosslinked Poly(Ethylene Glycol) Hydrogels,” Annals of Biomedical Engineering 32, no. 3 (2004): 407–417, 10.1023/B:ABME.0000017535.00602.ca. 15095815

[41] W. Li , D. Wang , W. Yang , and Y. Song , “Compressive mechanical properties and microstructure of PVA–HA hydrogels for cartilage repair,” RSC Advances 6, no. 24 (2016): 20166–20172, 10.1039/C6RA02166B.

[42] T. Sakai , T. Matsunaga , Y. Yamamoto , et al., “Design and Fabrication of a High‐Strength Hydrogel with Ideally Homogeneous Network Structure from Tetrahedron‐like Macromonomers,” Macromolecules 41, no. 14 (2008): 5379–5384, 10.1021/ma800476x.

[43] J. P. Gong , Y. Katsuyama , T. Kurokawa , and Y. Osada , “Double‐Network Hydrogels with Extremely High Mechanical Strength,” Advanced Materials 15, no. 14 (2003): 1155–1158, 10.1002/adma.200304907.

[44] B. Kang , Q. L. Lang , J. Tu , et al., “Preparation and properties of double network hydrogel with high compressive strength,” Polymers 14, no. 5 (2022): 966, 10.3390/polym14050966. 35267788 PMC8912320

[45] D. L. Butler , E. S. Grood , F. R. Noyes , R. Zernicke , and K. Brackett , “Effects of structure and strain measurement technique on the material properties of young human tendons and fascia,” Journal of Biomechanics 17, no. 8 (1984): 579–596, 10.1016/0021-9290(84)90090-3. 6490671

[46] K. J. Blose , J. E. Pichamuthu , J. S. Weinbaum , and D. A. Vorp , “Design and Validation of a Vacuum Assisted Anchorage for the Uniaxial Tensile Testing of Soft Materials,” Soft Materials 14, no. 2 (2016): 72–77, 10.1080/1539445X.2016.1141787. 27795696 PMC5082747

[47] M. Jiang , Z. T. Lawson , V. Erel , et al., “Clamping soft biologic tissues for uniaxial tensile testing: A brief survey of current methods and development of a novel clamping mechanism,” Journal of the Mechanical Behavior of Biomedical Materials 103 (2020): 103503, 10.1016/j.jmbbm.2019.103503. 32090940

[48] M. Gaplovsky , Y. V. Il'ichev , Y. Kamdzhilov , et al., “Photochemical reaction mechanisms of 2‐nitrobenzyl compounds: 2‐Nitrobenzyl alcohols form 2‐nitroso hydrates by dual proton transfer,” Photochemical & Photobiological Sciences 4, no. 1 (2005): 33–42, 10.1039/B409927C. 15616689

[49] Y. V. Il'ichev , M. A. Schwörer , and J. Wirz , “Photochemical Reaction Mechanisms of 2‐Nitrobenzyl Compounds: Methyl Ethers and Caged ATP,” Journal of the American Chemical Society 126, no. 14 (2004): 4581–4595, 10.1021/ja039071z. 15070376

[50] F. V. Lavrentev , V. V. Shilovskikh , V. S. Alabusheva , et al., “Diffusion‐Limited Processes in Hydrogels with Chosen Applications from Drug Delivery to Electronic Components,” Molecules 28, no. 15 (2023): 5931, 10.3390/molecules28155931. 37570901 PMC10421015

