# Supplementary material for: Rapid Photogelation of Low‐Concentration Polymer Solutions via Photodimerization of o‐Ethylnitrobenzene
Source: Adv Sci (Weinh). 2026 Aug 31:e77119. Online ahead of print. doi: 10.1002/advs.77119 (PMC13530358; doi:10.1002/advs.77119)
Supplement: Supplementary file 1 [file ADVS-9999-e77119-s008.pdf]

Supporting Information

Rapid Photogelation of Low-Concentration Polymer Solutions via Photodimerization of o-Ethylnitrobenzene

*Masaaki Okihara, Ayaka Tomoda, Kana Morishita, Toshiyuki Takagi, and Kimio Sumaru**

Cellular and Molecular Biotechnology Research Institute,
National Institute of Advanced Industrial Science and Technology
E-mail: k.sumaru@aist.go.jp

This PDF file includes:

Figure S1 to S13

Table S1 and S2

Captions for Movie S1 to S7

Other Supplementary Materials for this manuscript includes the following:

Movie S1 to S7

Table S1. Comparison of representative gelation mechanisms for polymer solutions (crosslinking) with respect to gelation trigger, gelation time, temporal and spatial controllability, scalability for bulk gel formation, and bioorthogonality during gelation.

	This work (ENB-mediated photocrosslinking)	Conventional photocrosslinking	Click chemistry	Condensation reaction	Enzymatic crosslinking
Gelation trigger	Light	Light	Mixing	Mixing	Mixing
Gelation time	+++ (0.5 s – 10 s)	++ (10 s – 10 min)	+++ (1 s – 10 min)	+ (1 min – several h)	++ (10 s – 10 min)
Temporal control	+++	+++	+	+	+
Spatial control	+++ (μm –)	+++ (μm –)	+	+	+
Gel size limitation*	++ (< a few cm)	+ (< a few mm)	+++	+++	+++
Bioorthogonality during gelation**	+++	+++	+++	+	++

* Gel size limitation is qualitatively evaluated considering limitations arising from light penetration or diffusion during gel formation.

** Bioorthogonality during gelation is qualitatively evaluated based on the possibility of selective crosslinking under physiological conditions without undesired reactions with naturally occurring biological molecules.

+++, excellent; ++, good; +, moderate. The values shown are representative ranges reported in the literature and may vary depending on the specific chemical systems and reaction conditions.

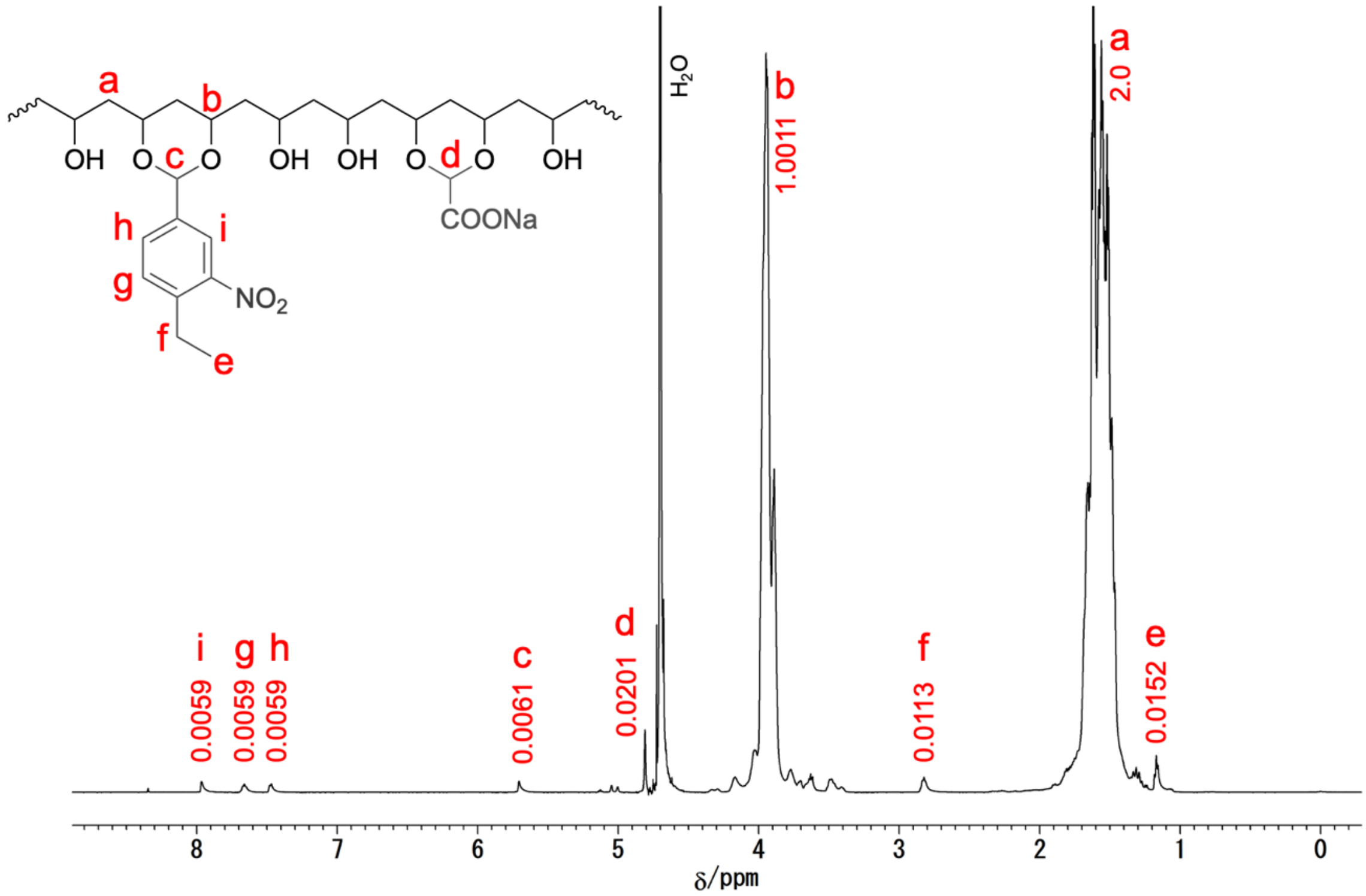

Figure S1. ^{1}H NMR spectra of ENBPVA (in D_2O, 64 times, 600 MHz).

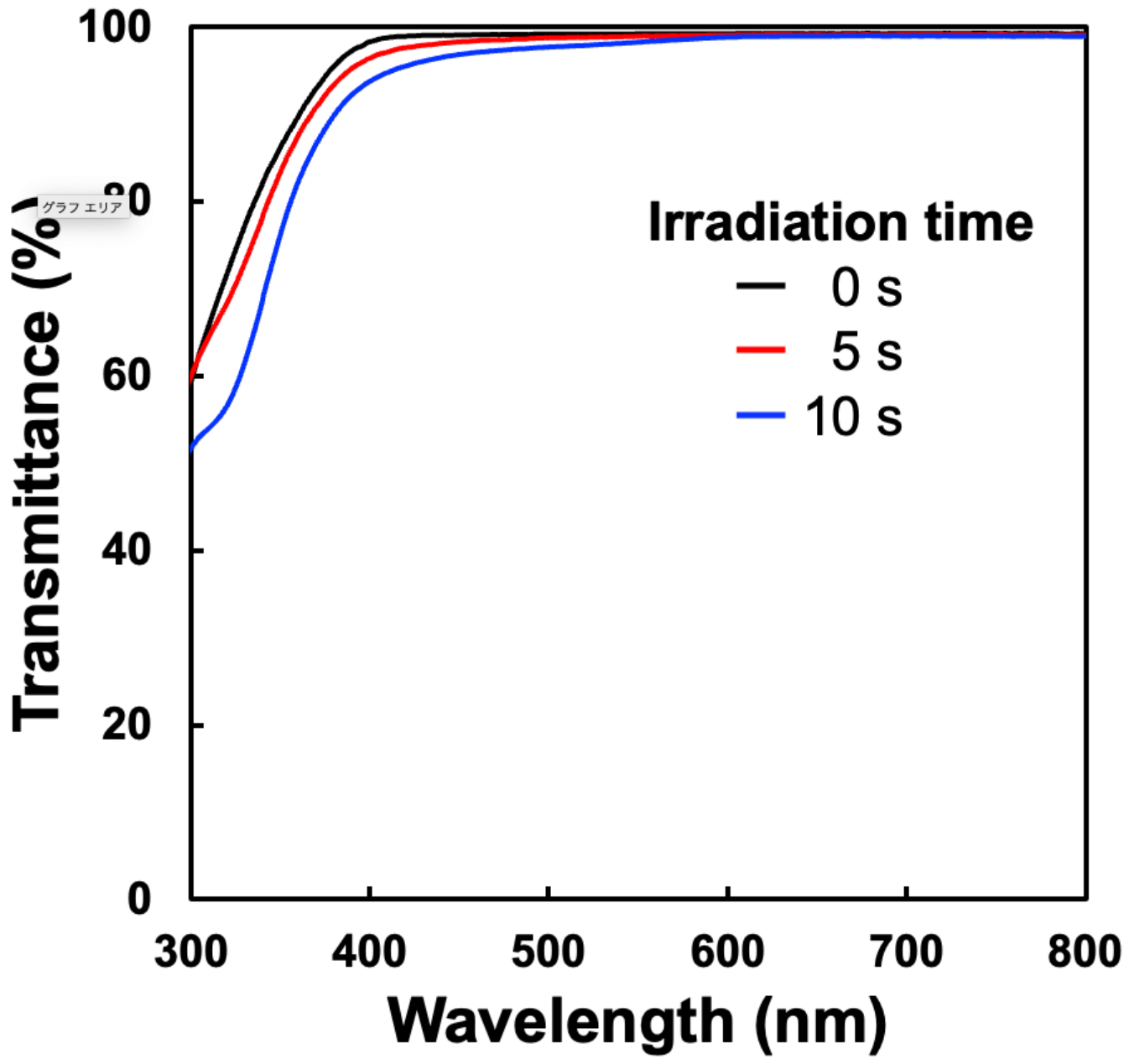

Figure S2. The transmittance of a 1 wt% ENBPVA aqueous solution before and after 5 and 10 seconds of UV irradiation (wavelength: 365 nm, intensity: 240 mW cm^{-2}). The optical path length was 1 mm.

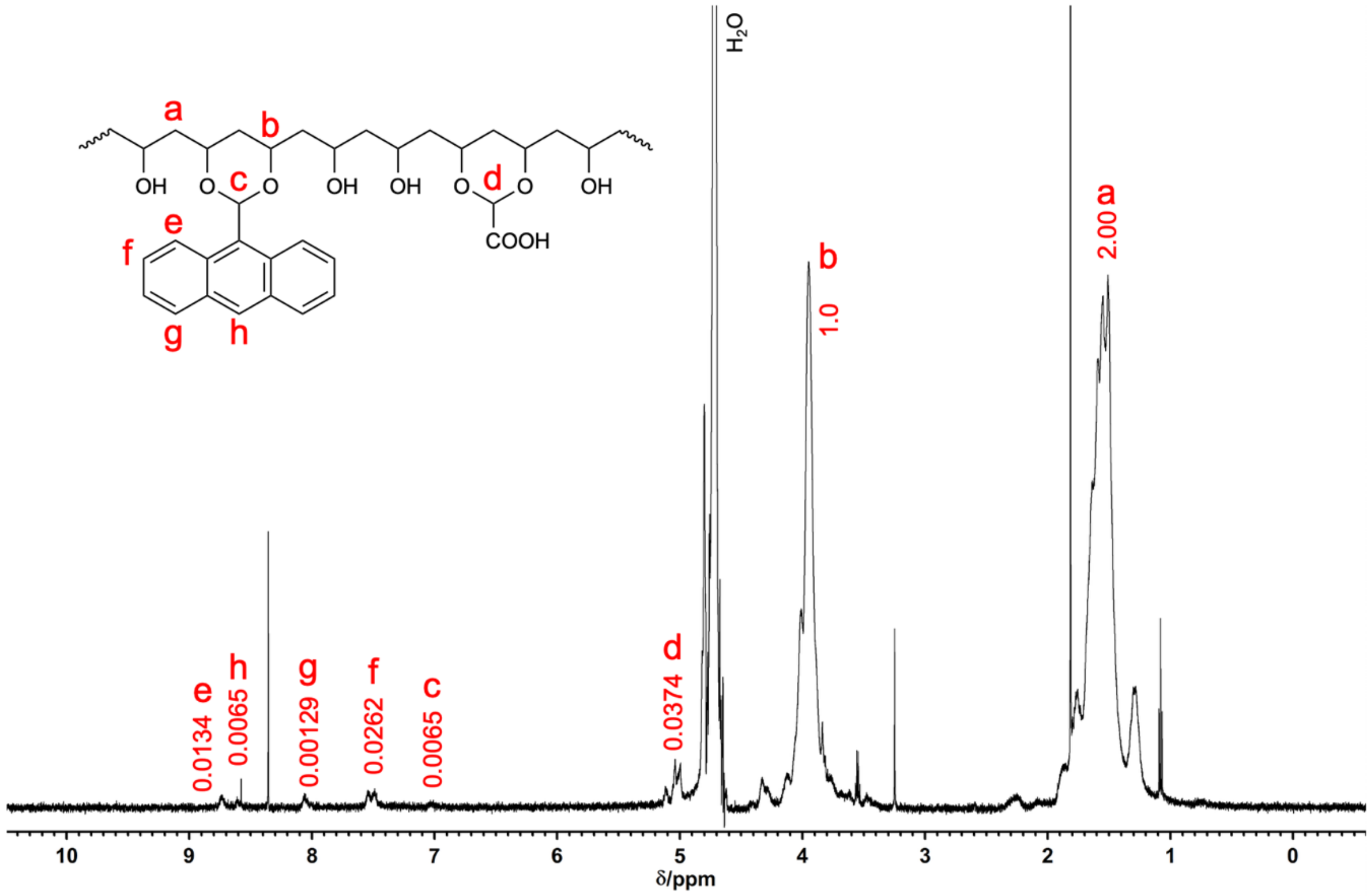

Figure S3. ^{1}H NMR spectra of AnthPVA (in D_2O, 64 times, 600 MHz).

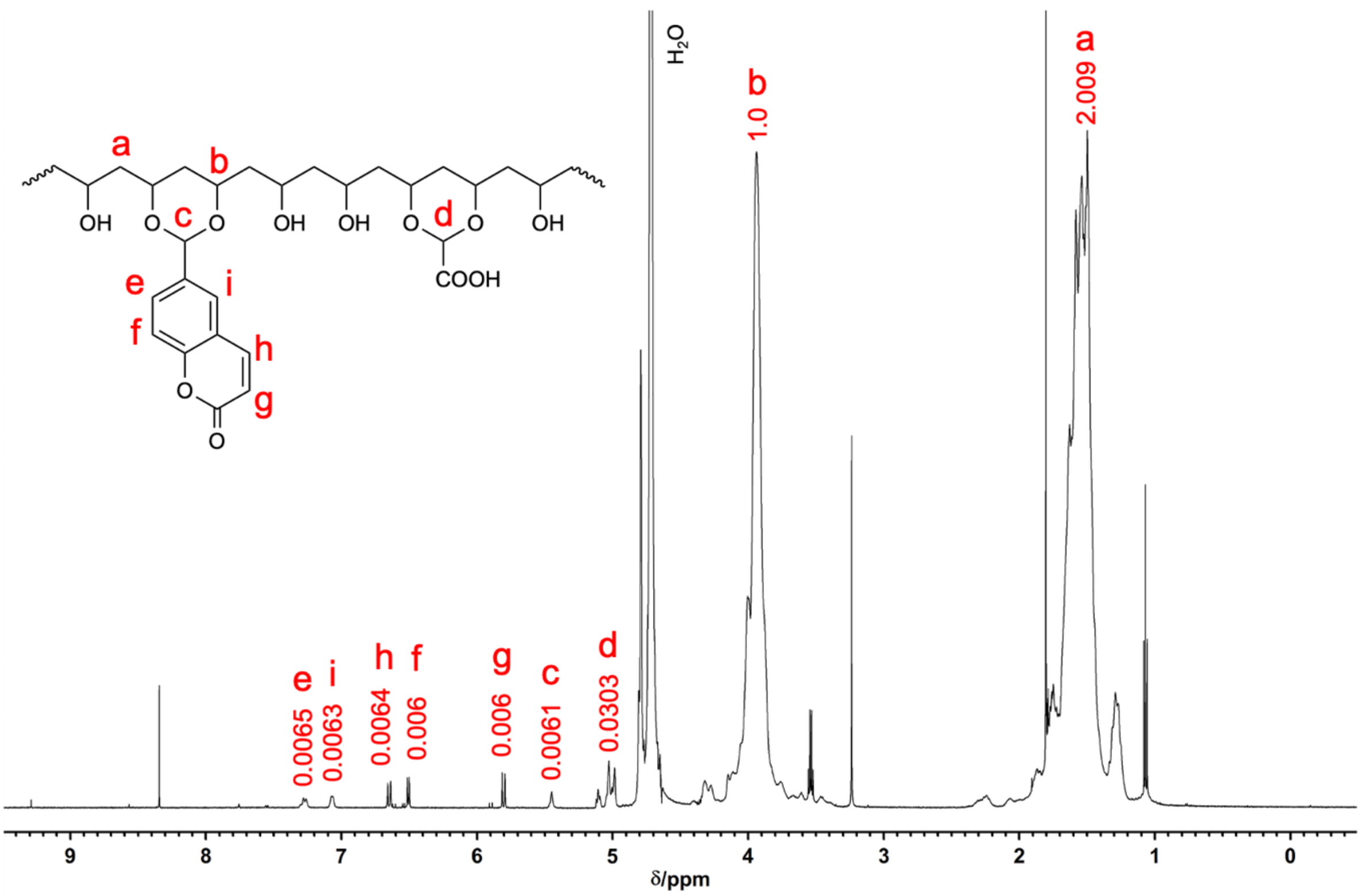

Figure S4. ^{1}H NMR spectra of CumPVA (in D_2O, 64 times, 600 MHz).

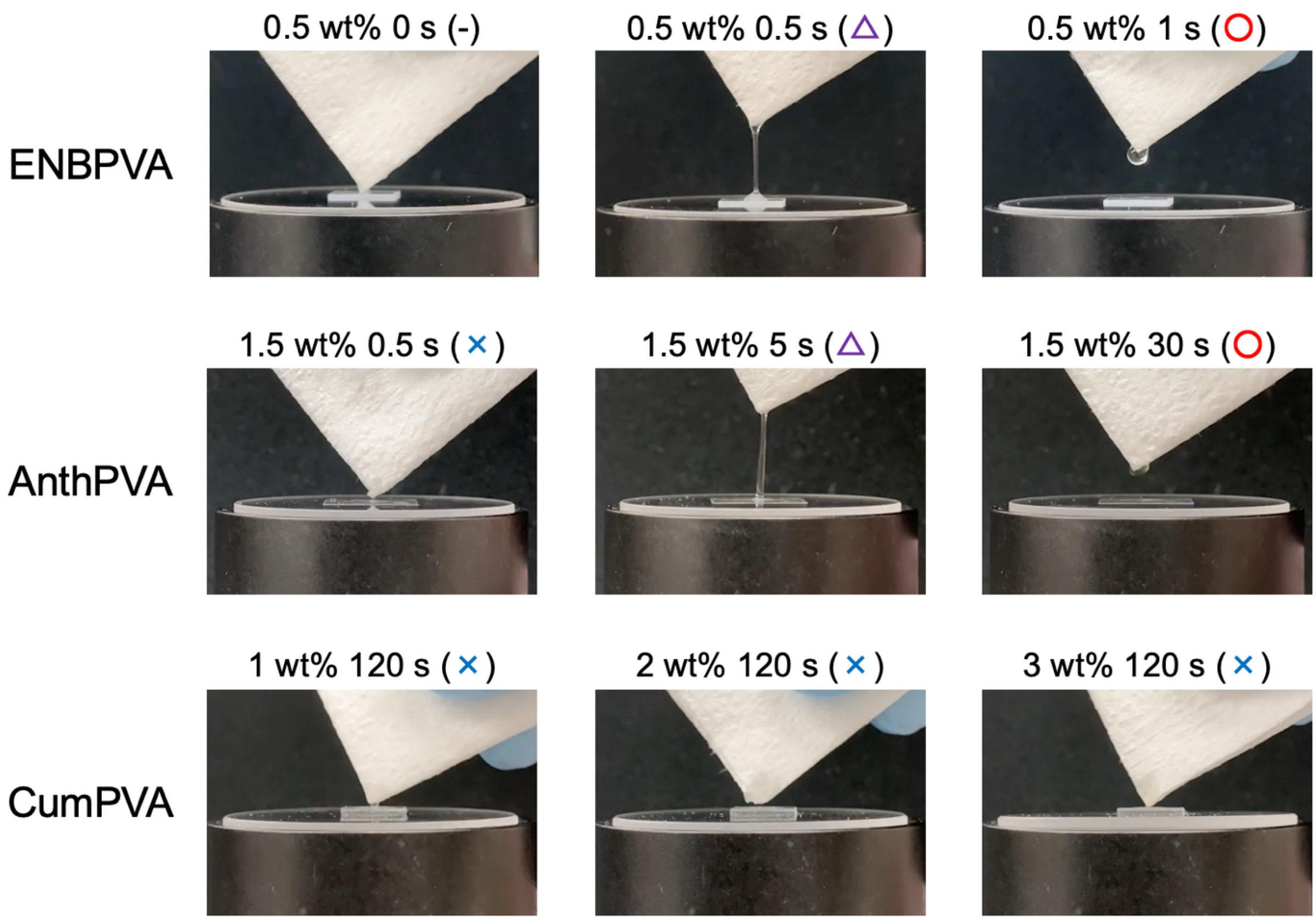

Figure S5. Representative images showing the behavior of aqueous solutions of ENBPVA, AnthPVA, and CumPVA upon contact with a wipe under selected conditions extracted from the phase diagrams (Figure 3). The corresponding polymer concentrations and irradiation times are indicated in each panel. The symbols denote sol (×), viscous sol (△), and gel (○) states.

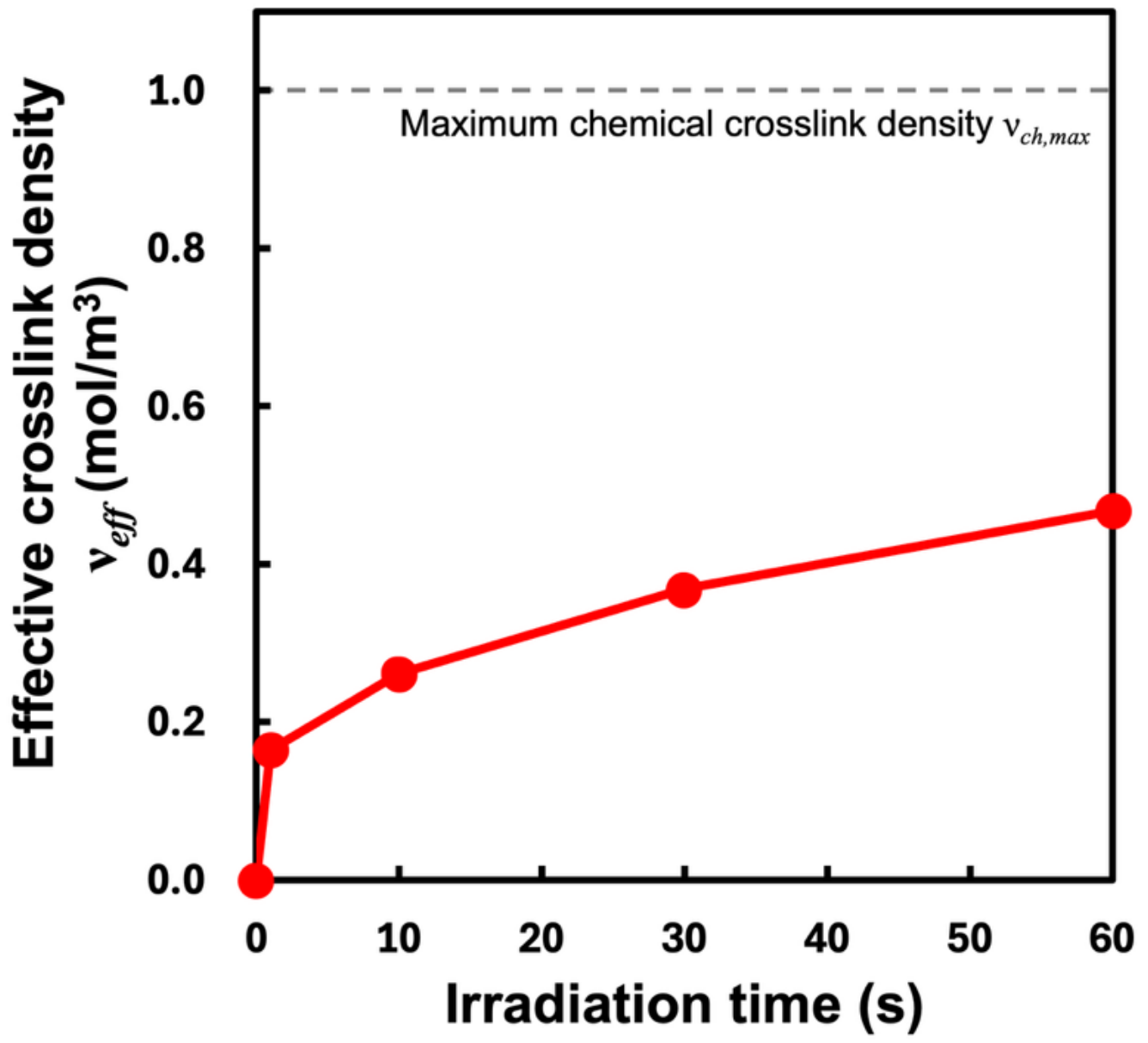

Figure S6. Influence of UV (wavelength: 365 nm, intensity: 240 mW cm^{-2}) irradiation time on effective crosslink density (ν_{eff}) calculated from elastic moduli of 1.5 wt% ENBPVA hydrogels. The maximum chemical crosslink density was calculated to be 1 mol/m^3 from the concentration of ENB groups contained in the solution ([ENB]/2).

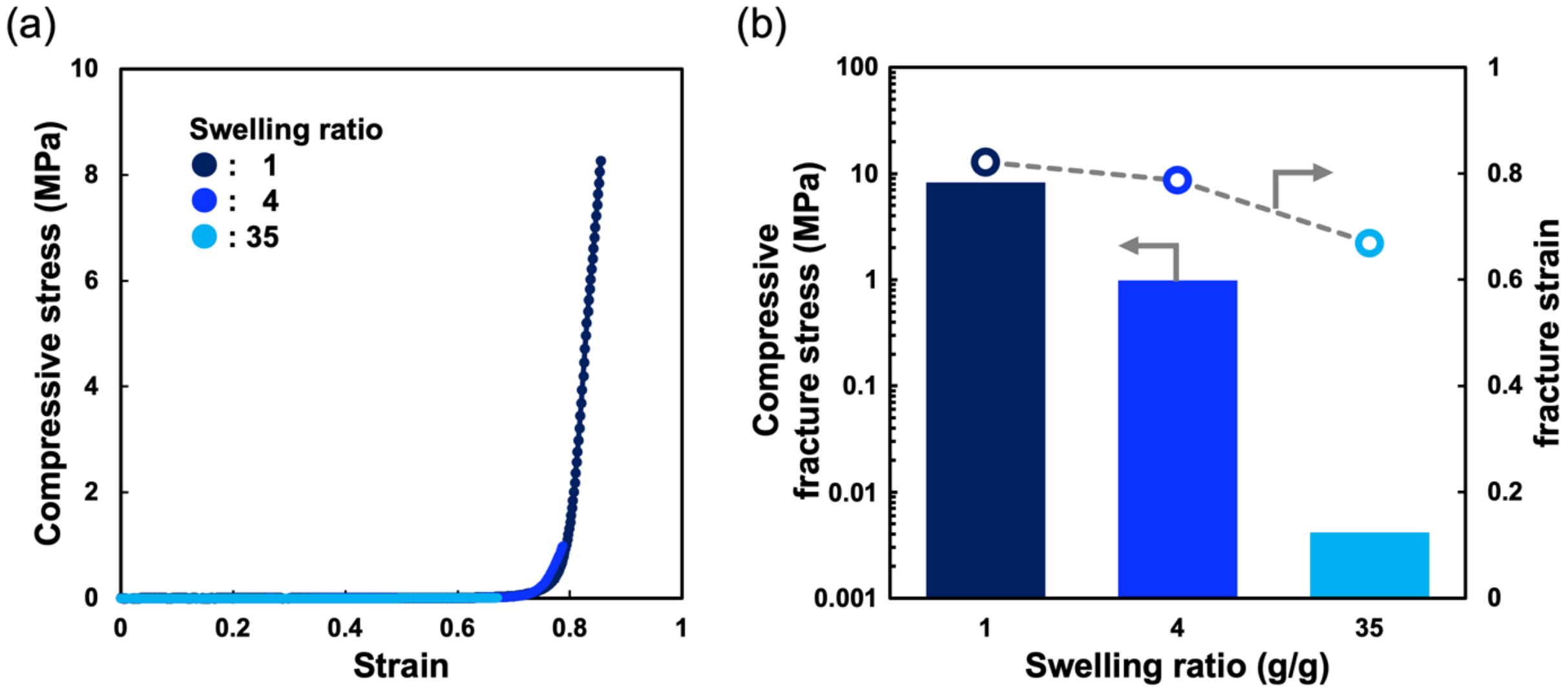

Figure S7. (a) Stress–strain curves of 2 wt% ENBPVA hydrogels prepared by 10 seconds of UV irradiation (365 nm, 240 mW cm^{-2}) and subsequently immersed in ultrapure water to reach predefined swelling ratios. (b) Fracture compressive stress and strain of the swollen ENBPVA hydrogels, extracted from the stress–strain curves in Figure S4a, plotted as a function of swelling ratio.

Table S2. List of polymer concentrations and compressive fracture stresses of previously reported hydrogels and ENBPVA hydrogels (in this work).

	Types of hydrogels	Main components of hydrogel	Polymer Concentration (wt%)	Fracture Compressive Stress (kPa)	Reference [Citation number]
1	Natural polymer-based hydrogels	Gelatin	7	1	*Mater. Des.* **183** (2019): 108113 [33]
2	Natural polymer-based hydrogels	Collagen	1	3	*J. Mech. Behav. Biomed. Mater.* **2**(2) (2009): 202–209 [34]
3	Natural polymer-based hydrogels	Hyaluronic acid methacrylate	2	10.2	*Biomed. Mater.* **17**(2) (2022): 025006 [35]
4	Synthetic polymer hydrogels	PVA	7	20	*Mater. Des.* **183** (2019): 108113 [33]
5	Natural polymer-based hydrogels	Chitosan	2	25.9	*Cellulose* **24** (2017): 2215–2228 [36]
6	Natural polymer-based hydrogels	Gelatin methacrylate	1.57	35	*Cellulose* **24** (2017): 2215–2228 [36]
7	Natural polymer-based hydrogels	Agarose	1	38	*Biomacromolecules* **1**(4) (2000): 730–738 [37]
8	Natural polymer-based hydrogels	Alginate	2	40	*Biomaterials* **26** (2005): 2455–2465 [38]
9	Synthetic polymer hydrogels	PAA	1	100	*Adv. Mater.* **15** (2003): 1155–1158 [43]
10	Natural polymer-based hydrogels	Gelatin methacrylate	5.7	210	*J. Nanobiotechnol.* **19** (2021): 275 [39]
11	Natural polymer-based hydrogels	Collagen	7	260	*Adv. Mater.* **15** (2003): 1155–1158 [43]
12	Synthetic polymer hydrogels	PEGDA	20	670	*Ann. Biomed. Eng.* **32** (2004): 407–417 [40]
13	Synthetic polymer hydrogels	PAAm	7	700	*Adv. Mater.* **15** (2003): 1155–1158 [43]
14	Synthetic polymer hydrogels	PVA-Hydroxyapatite	6	840	*RSC Adv.* **6** (2016): 20166–20172 [41]
15	Natural polymer-based hydrogels	Agarose	5	929	*Biomacromolecules* **1**(4) (2000): 730–738 [37]
16	Double network hydrogels	Cellulose/Gelatin	22	3700	*Adv. Mater.* **15** (2003): 1155–1158 [43]
17	High-strength hydrogels	PAAm	15.1	6000	*NPG Asia Mater.* **13** (2021): 34 [32]
18	High-strength hydrogels	Tetra-PEG	10.7	9600	*Macromolecules* **41** (2008): 5379–5384. [42]
19	Double network hydrogels	PAMPS/PAAm	10	17200	*Adv. Mater.* **15** (2003): 1155–1158 [43]
20	Double network hydrogels	P(AMPS-co-TFEA)/PAAm	7	21000	*Adv. Mater.* **15** (2003): 1155–1158 [43]
21	Double network hydrogels	P(AMPS-co-AM)/PAM	19	37800	*Polymer* **14** (2022): 966 [44]
This work	Synthetic polymer hydrogels	PVA	1	2550	-
			2	8270	
			3	10550	

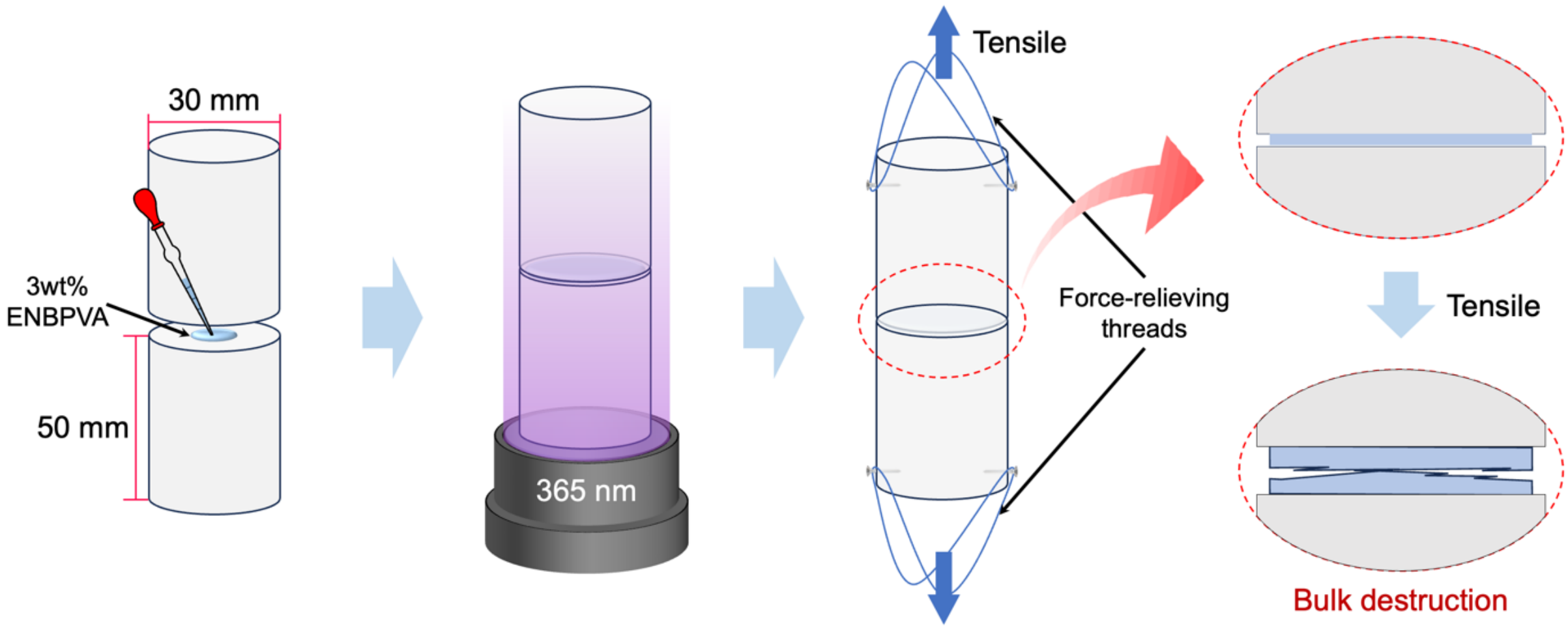

Figure S8. Schematic illustration showing the adhesion test: A drop of 3 wt% ENBPVA aqueous solution was placed between two transparent acrylic resin cylinders (height: 50 mm, diameter: 30 mm) with strings fixed. Then the sample was irradiated with UV light (wavelength: 365 nm, intensity: 240 mW cm^{-2}) from below to induce gelation of the solution. Adhesive strength was evaluated either by tensile testing or by lifting a 12 kg chair.

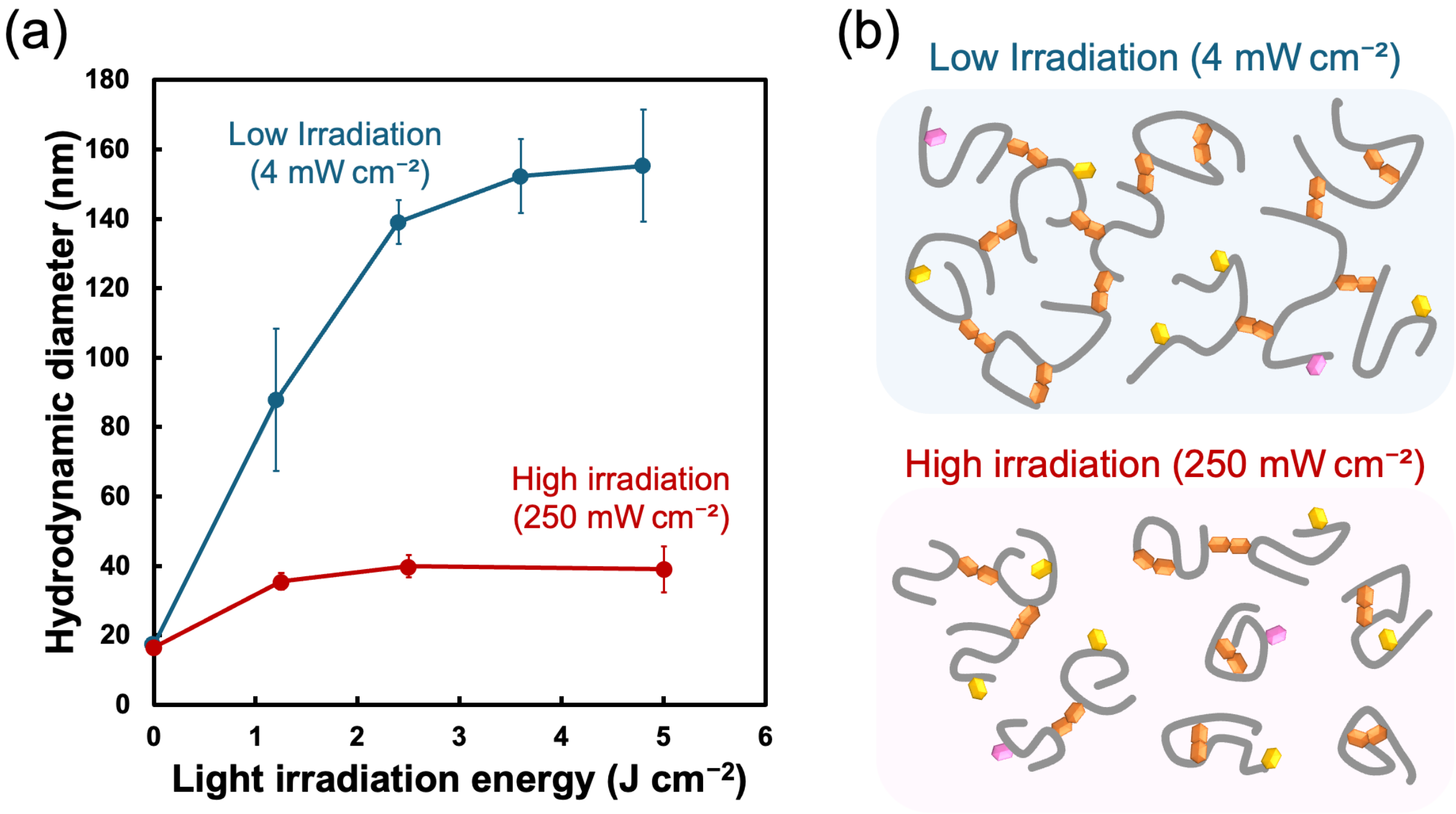

Figure S9. (a) Influence of the UV irradiation condition (wavelength: 365 nm, intensities: 250 and 4 mW cm^{-2}) on hydrodynamic diameter of ENBPVA in a 0.2 wt% aqueous solution (below the gelation threshold), measured by dynamic light scattering (DLS). (b) Schematic illustration of the effect of varying light intensities on the photo-crosslinking behavior of ENBPVA. Preferential interpolymer crosslinking promotes under low irradiation intensity.

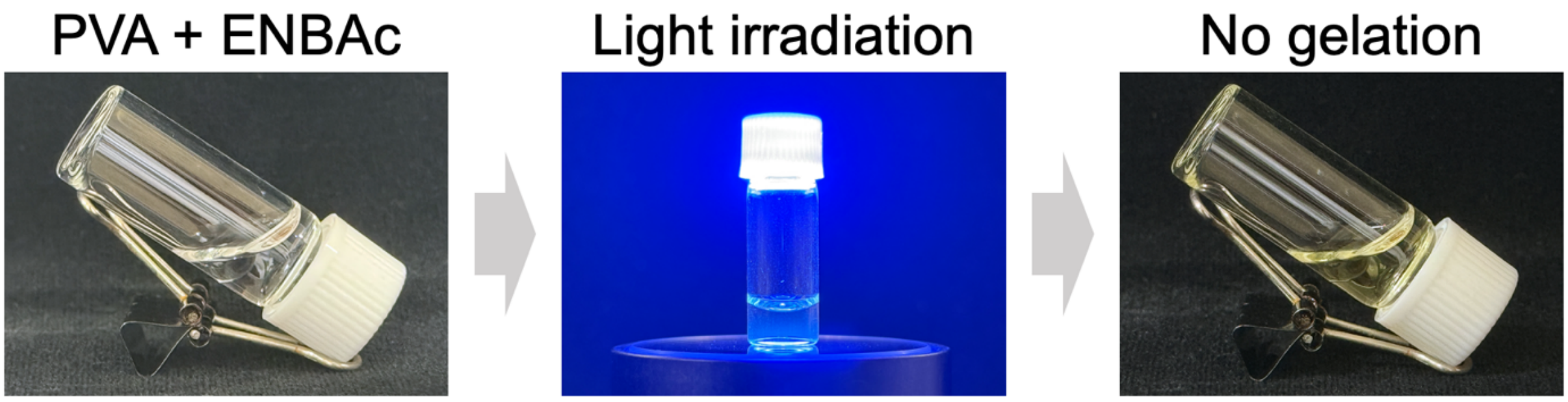

Figure S10. Sequential photographs of an aqueous mixture of poly(vinyl alcohol) (PVA) and 4-ethyl-3-nitrobenzoic acid (ENBAc), a water-soluble small molecule bearing ENB groups, before, during, and after UV irradiation (365 nm, 240 mW cm^{-2}, 30 s).

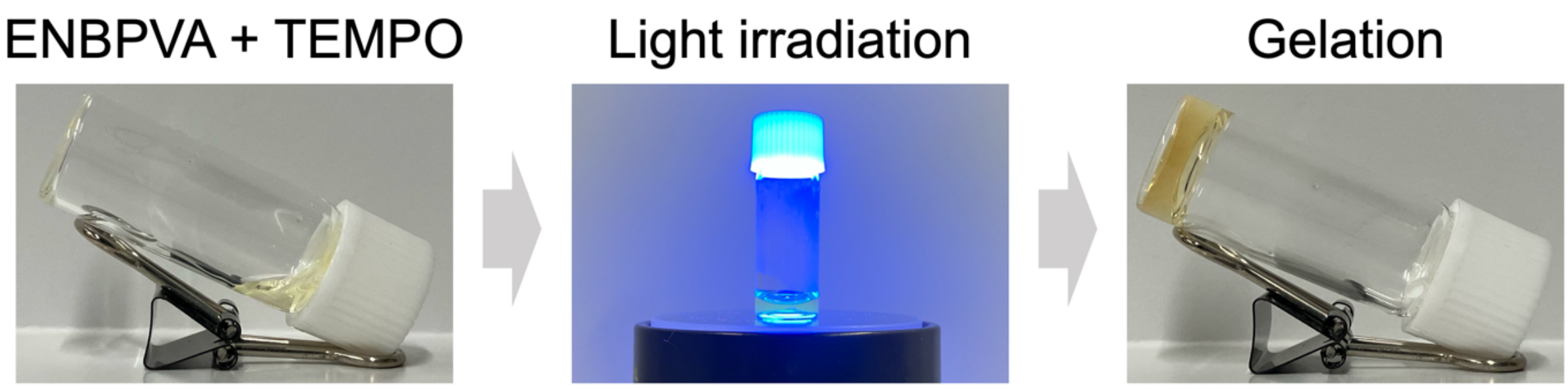

Figure S11. Sequential photographs of a 2 wt% ENBPVA aqueous solution containing TEMPO (5 wt%), a radical scavenger, taken before, during, and after UV irradiation (365 nm, 240 mW cm^{-2}, 10 s) to evaluate the effect of TEMPO on photogelation.

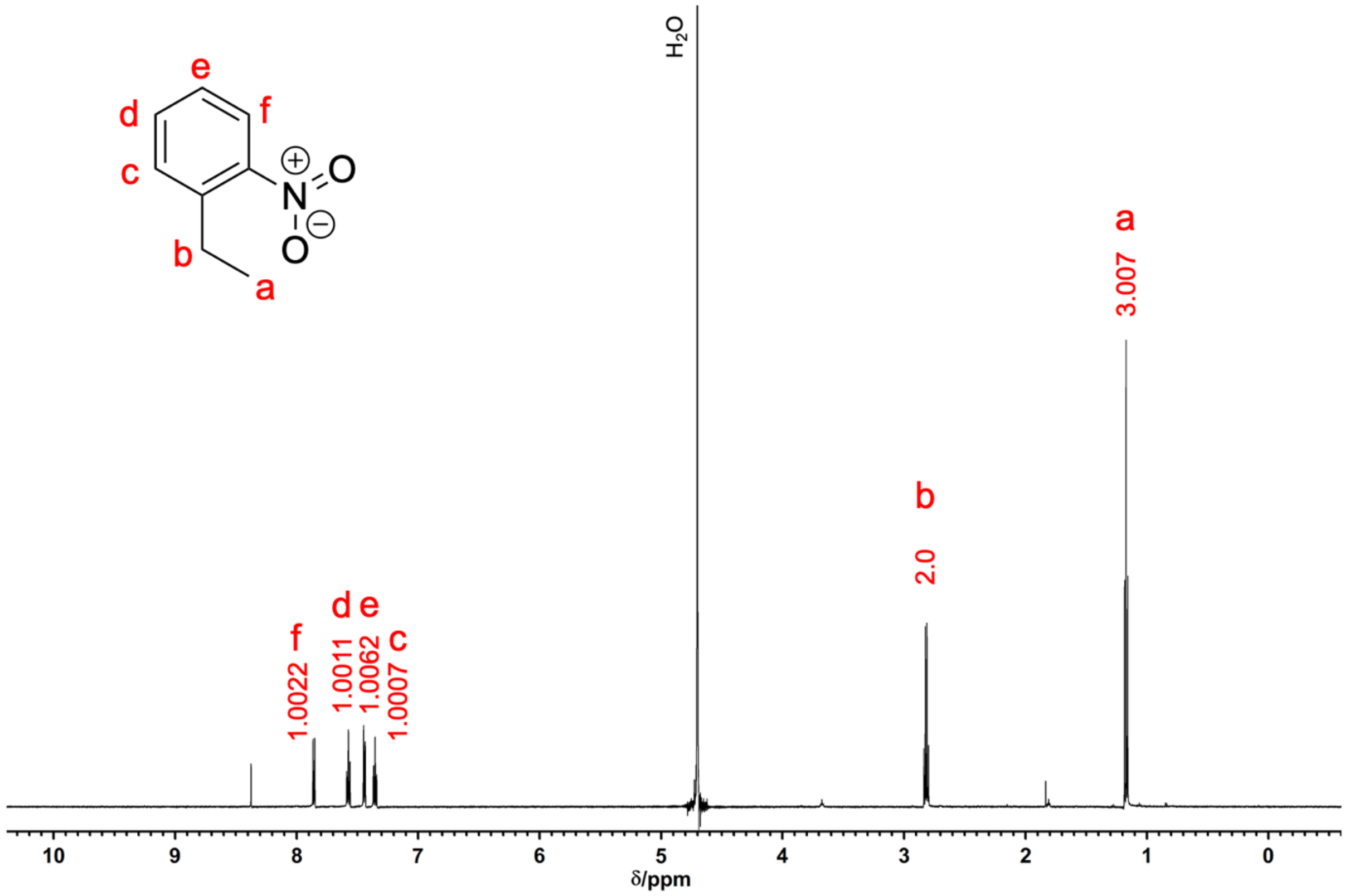

Figure S12. ^{1}H NMR spectra of 2-Ethylnitrobenzene (in D_2O, 64 times, 600 MHz).

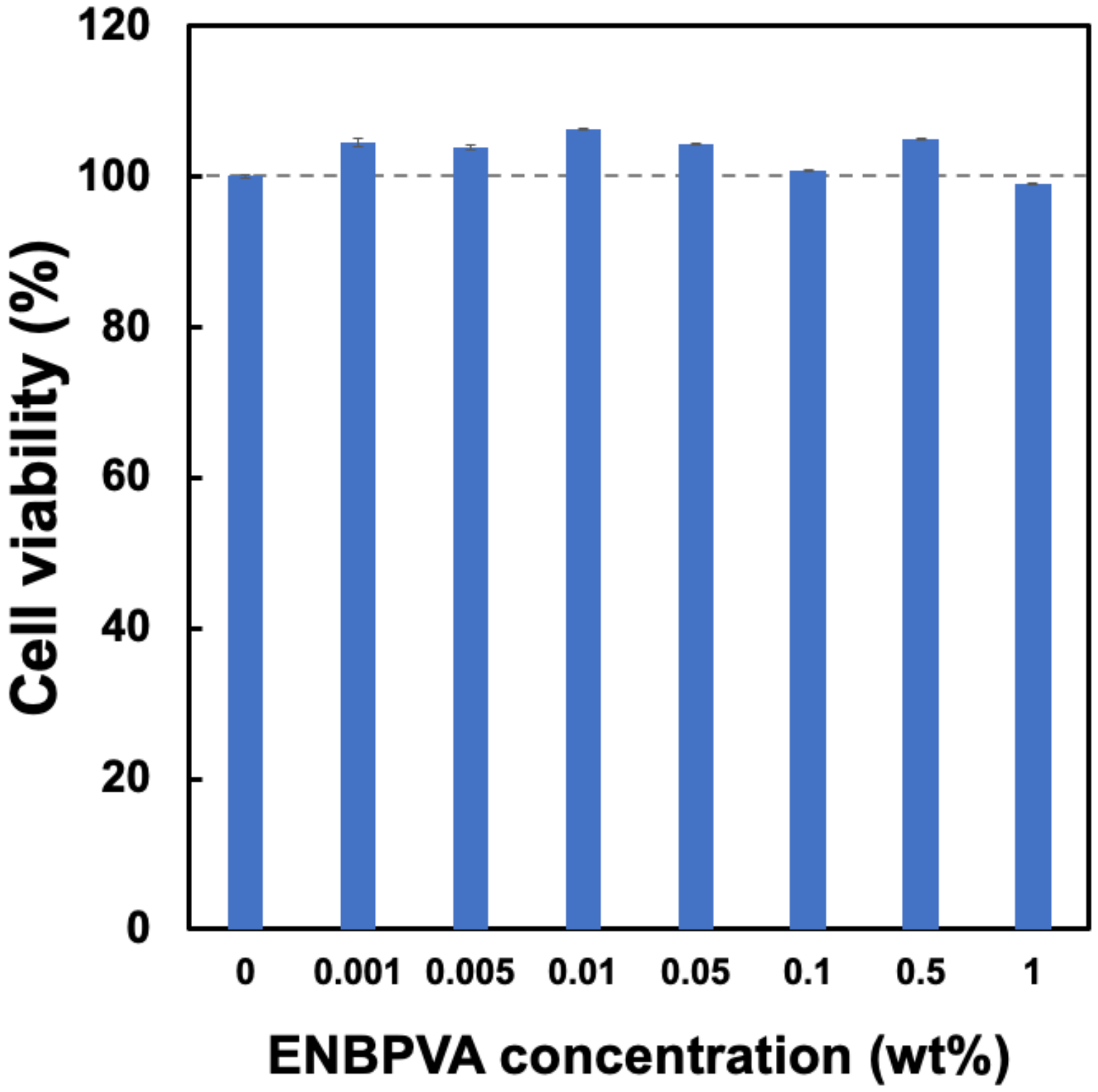

Figure S13. Cell viability of mouse fibroblast 3T3 cells cultured for 1 day in media containing various concentrations of ENBPVA, as assessed by the WST-8 assay. Viability in control medium without polymer was defined as 100%.

Captions for Supplementary Movies

Movie S1. Gelation behavior of a 2 wt% ENBPVA solution upon irradiation with 365 nm UV light for 5 seconds.

Movie S2. Time-lapse movie showing the swelling of dried, bear-shaped hydrogels immersed in ultrapure water.

Movie S3. Process of photo-induced formation (5 seconds) and swelling (1 day) of ENBPVA hydrogels prepared from a 2 wt% aqueous solution.

Movie S4. Demonstration showing the mechanical property of hydrogels prepared by irradiating a 3 wt% ENBPVA aqueous solution with 365 nm UV light for 0.5 seconds.

Movie S5. Movie of a 12 kg chair being lifted by hand using two cylindrical acrylic rods bonded with ENBPVA hydrogel as connectors. The rods were bonded by sandwiching a 3 wt% ENBPVA solution between them, followed by UV irradiation (365 nm, 240 mW cm^{-2}) for 40 s.

Movie S6. Evaluation of the tensile strength of 3 wt% ENBPVA hydrogel bonding two cylindrical acrylic resins using uniaxial tensile testing.

Movie S7. Demonstration showing that ENBPVA photo-crosslinking is due to dimer formation between ENB groups. Test samples were 1.5 wt% ENBPVA aqueous solution only (left), containing benzoic acid (center), and ENBPVA with ENBAc (right). The amounts of coexisting ENBAc or benzoic acid were adjusted to be equal to that of ENB groups incorporated in ENBPVA. After UV irradiation (365 nm, 240 mW cm^{-2}) for 20 seconds, gelation of each sample was checked by the observation of soak-in behavior on a paper wiper.
